# TRPV4 blockade suppresses atrial fibrillation in sterile pericarditis rats

**DOI:** 10.1172/jci.insight.137528

**Published:** 2020-12-03

**Authors:** Jie Liao, Qiongfeng Wu, Cheng Qian, Ning Zhao, Zhaoyang Zhao, Kai Lu, Shaoshao Zhang, Qian Dong, Lei Chen, Qince Li, Yimei Du

**Affiliations:** 1 Department of Cardiology,; 2 Research Center of Ion Channelopathy,; 3 Institute of Cardiology, and; 4 Key Lab for Biological Targeted Therapy of Education Ministry and Hubei Province, Union Hospital, Tongji Medical College, Huazhong University of Science and Technology, Wuhan, China.; 5 Department of Physiology, Nanjing Medical University, Nanjing, China.; 6 Harbin Institute of Technology, Nangang District, Harbin, China.

**Keywords:** Cardiology, Arrhythmias, Calcium, Fibrosis

## Abstract

Atrial fibrillation (AF) commonly occurs after surgery and is associated with atrial remodeling. TRPV4 is functionally expressed in the heart, and its activation affects cardiac structure and functions. We hypothesized that TRPV4 blockade alleviates atrial remodeling and reduces AF induction in sterile pericarditis (SP) rats. TRPV4 antagonist GSK2193874 or vehicle was orally administered 1 day before pericardiotomy. AF susceptibility and atrial function were assessed using in vivo electrophysiology, ex vivo optical mapping, patch clamp, and molecular biology on day 3 after surgery. TRPV4 expression increased in the atria of SP rats and patients with AF. GSK2193874 significantly reduced AF vulnerability in vivo and the frequency of atrial ectopy and AF with a reentrant pattern ex vivo. Mechanistically, GSK2193874 reversed the abnormal action potential duration (APD) prolongation in atrial myocytes through the regulation of voltage-gated K^+^ currents (*I*_K_); reduced the activation of atrial fibroblasts by inhibiting P38, AKT, and STAT3 pathways; and alleviated the infiltration of immune cells. Our results reveal that TRPV4 blockade prevented abnormal changes in atrial myocyte electrophysiology and ameliorated atrial fibrosis and inflammation in SP rats; therefore, it might be a promising strategy to treat AF, particularly postoperative AF.

## Abstract 2

TRPV4 blockade prevents abnormal changes in atrial myocyte electrophysiology and ameliorated atrial fibrosis in rats and might be a promising strategy to treat atrial fibrillation.

## Graphical abstract

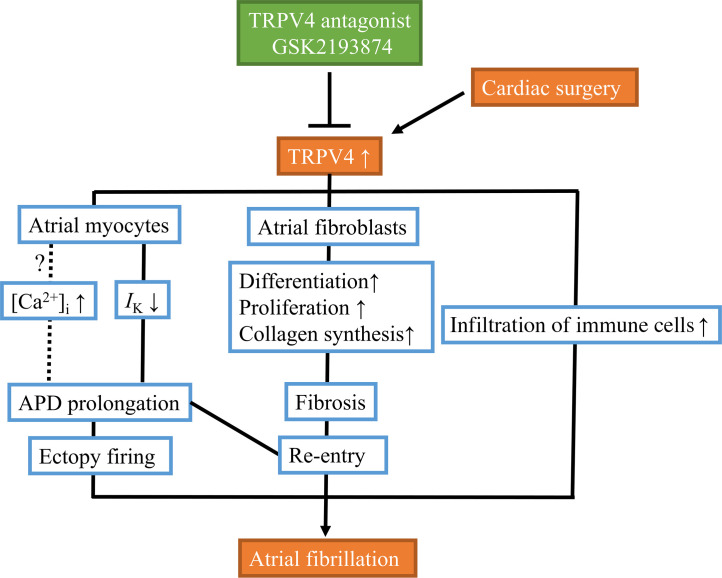

## Introduction

Atrial fibrillation (AF) is the most common sustained arrhythmia observed in clinical settings and often occurs after cardiac surgery (1). Atrial remodeling induced by inflammatory profibrotic signals plays a critical role in the pathogenesis of AF (2). For instance, cardiac surgery stimulates inflammatory profibrotic signals and subsequently leads to enhanced vulnerability to AF in patients (3) as well as in the sterile pericarditis (SP) animal model, a well-established model of human postoperative AF (4–6). The reduction of inﬂammation profibrotic signals and reversal of atrial remodeling may be potential therapeutic targets for the treatment of AF (7).

Transient receptor potential vanilloid 4 (TRPV4) is a Ca^2+^-permeable cation channel that can be activated by various physical and chemical stimuli, including heat, mechanical stretch, hypo-osmotic stimulation, and many inflammatory metabolites (8). TRPV4 is expressed in cardiomyocytes, fibroblasts, endothelial cells, and smooth muscle cells, as well as in tissues such as the lung, heart, liver, and skin (8). Our previous research found that the functional expression of TRPV4 elevates in the left ventricular tissue in ischemia/reperfusion models (9). In cardiomyocytes, TRPV4 activation leads to mPTP opening and cell apoptosis during hypoxia/reoxygenation (10). The blockade or deletion of TRPV4 reduces infarct sizes and improves cardiac function (9–12). In neurons, TRPV4 activation has been reported to modulate other ion currents, including voltage-gated K^+^ currents (*I*_K_), and to result in action potential (AP) alteration (13, 14). In cardiac fibroblasts, TRPV4 activation is required for TGF-β1–induced differentiation (15). Outside of the heart, the significance of TRPV4 has been implicated in many inflammatory fibrosis diseases, such as lung, liver, and skin fibrosis (16, 17). Cell apoptosis and fibrosis contribute notably to the loss of electrical conductivity between cardiomyocytes and, subsequently, the conduction disturbances in the fibrillating atria. Therefore, we hypothesize that the blockade of TRPV4 alleviates atrial remodeling and reduces the induction of AF.

To test this hypothesis, we first assessed TRPV4 expression changes in the atria of SP rats and AF patients. We then investigated the effect of TRPV4 antagonist GSK2193874 on the vulnerability to AF in vivo. The mechanisms of AF were examined by optical mapping. We also characterized the underlying signaling events in SP rats, as well as in isolated atrial myocytes and fibroblasts.

## Results

### The expression of TRPV4 increases in the atria of SP rats.

**Figure 1:**
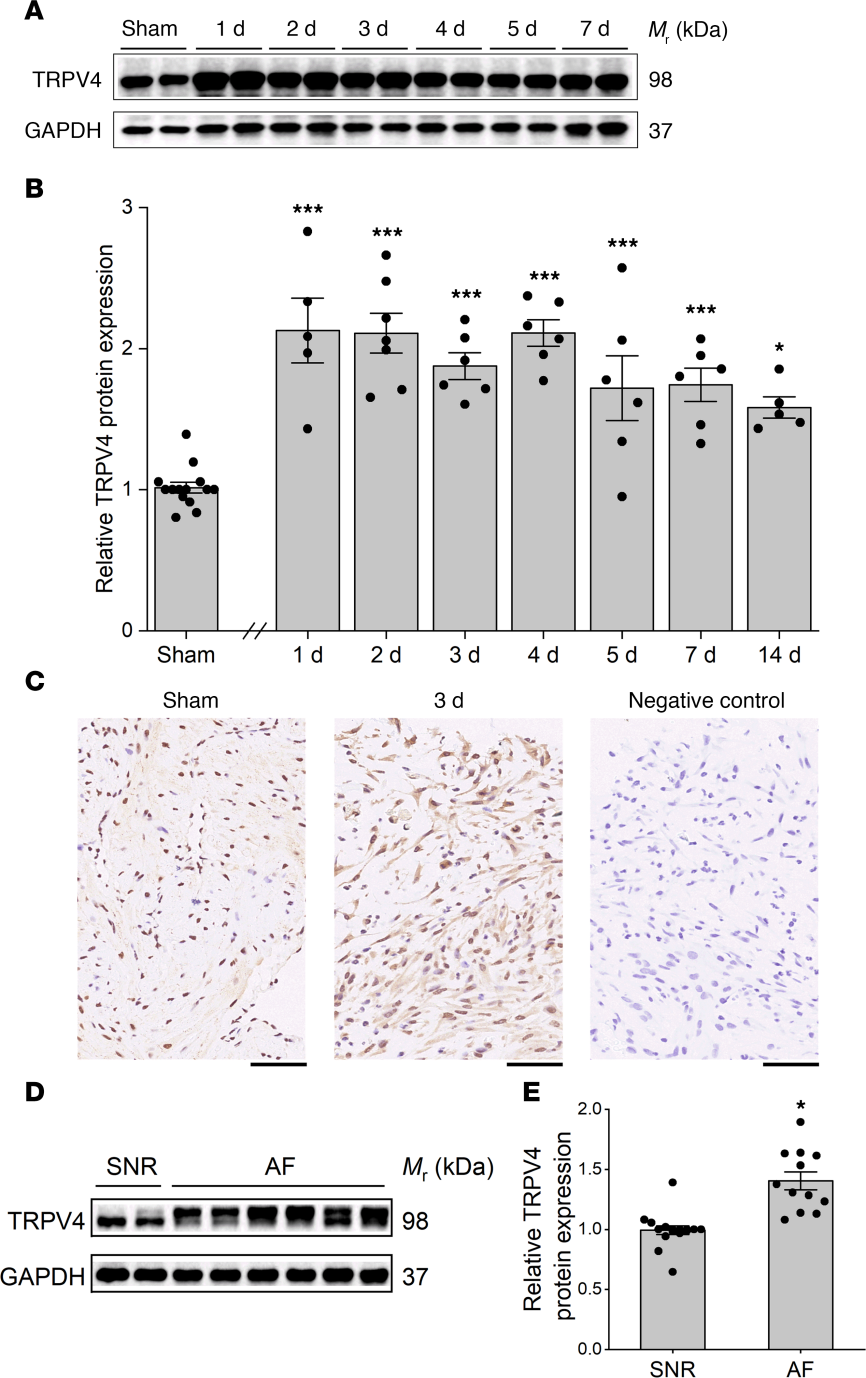
Upregulated expression of TRPV4 in the atria of SP rats and AF patients. (**A** and **B**) Representative Western blot (**A**) and quantification (**B**) of TRPV4 in atrial tissue of sham (*n* = 14) and SP rats 1 day (d) (*n* = 5), 2 d (*n* = 7), 3 d (*n* = 6), 4 d (*n* = 6), 5 d (*n* = 6), 7 d (*n* = 6), and 14 d (*n* = 5) after surgery. (**C**) The expression of TRPV4 in hearts was measured at day 3 after surgery using IHC. The negative control shown was treated using the same immunohistochemical procedure, but the primary antibody step was omitted. Scale bar: 50 μm. (**D** and **E**) Representative Western blot (**D**) and quantification (**E**) of TRPV4 in atrial tissue of patients with in sinus rhythm (SNR, *n* = 16) and AF (*n* = 12). Statistical analyses: 1-way ANOVA with Bonferroni’s post hoc test (**B**) and 2-tailed unpaired Student’s t test (**E**); *P < 0.05, ***P < 0.001. Data are expressed as the mean ± SEM.

To assess whether TRPV4 is involved in AF, we measured the time-dependent changes in the expression levels of TRPV4 in the atria after surgery. The protein expression of TRPV4 markedly increased on day 1 following surgery and then gradually decreased, but it still maintained a higher level on day 14 (Figure 1, A and B). This result was confirmed by immunohistochemical staining. As shown in Figure 1C, positive immunoreaction for TRPV4 was confined to the nuclear zone in sham-operated rats, but the signal was stronger and clearly visible on the outside of the nucleus on day 3. These results suggest that the expression of TRPV4 was upregulated in the atria of SP rats. To determine the cell type–specific TRPV4 expression patterns in atria, we performed costaining with TRPV4 and Cardiac troponin T (for cardiomyocytes) or vimentin (for fibroblasts) antibodies in atria (Supplemental Figure 1; supplemental material available online with this article; https://doi.org/10.1172/jci.insight.137528DS1), as well as in isolated atrial myocytes and atrial fibroblasts (Supplemental Figure 2). DAPI (blue) was used to stain the nucleus. TRPV4 immunostaining was found in both atrial myocytes and fibroblasts, and its staining intensity was much stronger in the vehicle group than the sham group. Note the number of vimentin-positive cells increased significantly. We repeated the above immunostaining using a second TRPV4 antibody (A5660, ABclonal) and obtained similar results (data not shown). We also assessed TRPV4 expression levels in atrial samples from cardiac surgery patients and found significant TRPV4 upregulation in AF patients relative to control patients in sinus rhythm (Figure 1, D and E, and Supplemental Table 1).

### TRPV4 antagonist GSK2193874 reduces susceptibility to AF.

**Figure 2:**
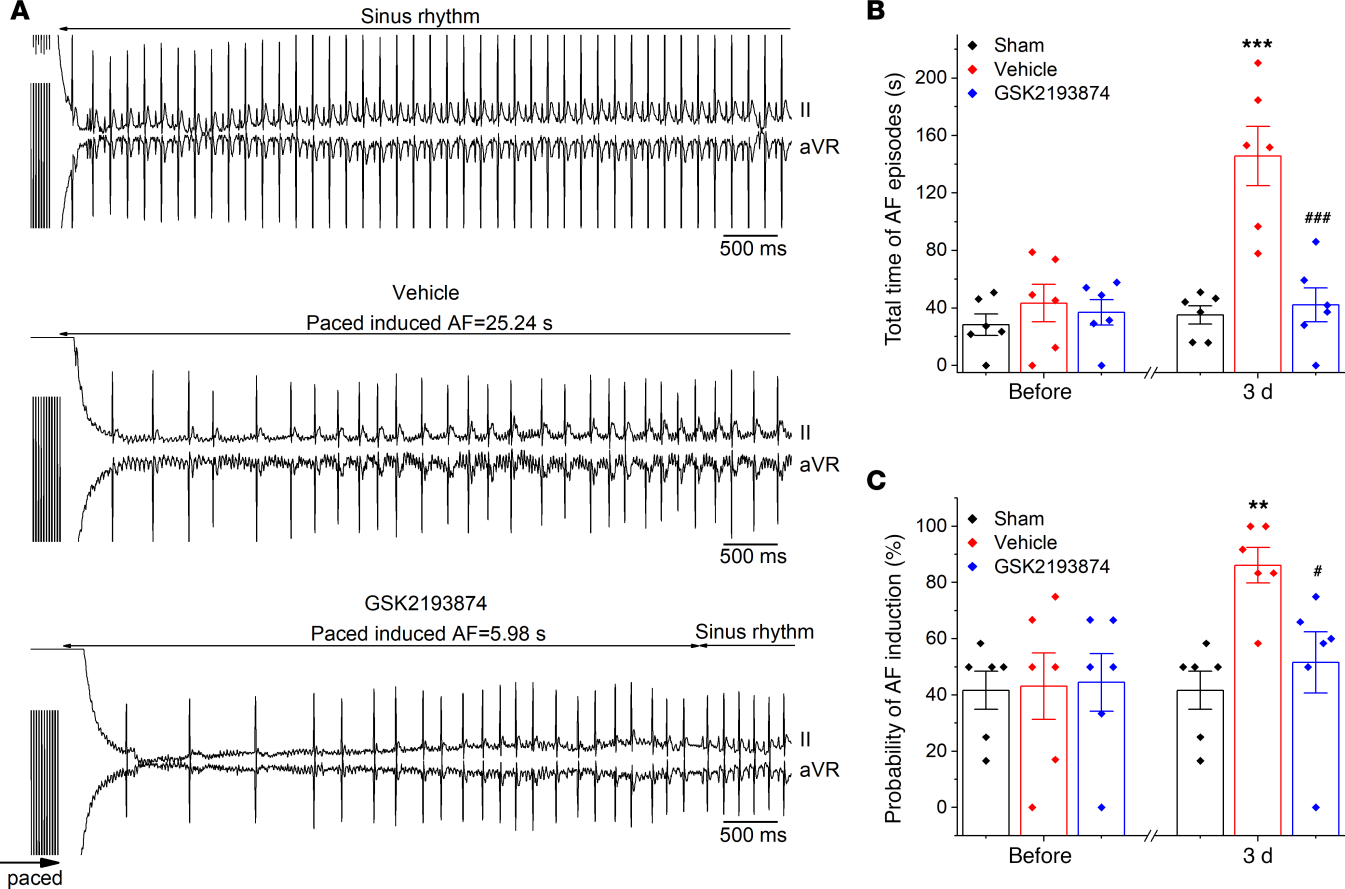
Blockage of TRPV4 suppresses atrial fibrillation induction and duration in sterile pericarditis rats. (**A**) Typical ECG recording results from the sham, vehicle, and GSK2193874 groups. (**B** and **C**) Statistical results of atrial fibrillation duration and probability of induced atrial fibrillation (AF) before and 3 d after operation among 3 groups. *n* = 6 each group. Statistical analyses: 1-way ANOVA with Bonferroni’s post hoc test (**B** and **C**); **P < 0.01, ***P < 0.001 versus sham; ^#^*P* < 0.05, ###*P* < 0.001 versus vehicle. Data are expressed as the mean ± SEM.

To further examine the role of TRPV4 in AF, we treated the SP rats with GSK2193874 (30 mg/kg/day) (18), an oral-specific TRPV4 blocker, 1 day before surgery. Supplemental Table 2 presents the electrophysiological characteristics of all groups. No significant differences were observed in the parameters examined either before surgery or 3 days after surgery. Figure 2A shows representative electrocardiograms (ECG) during the AF-inducibility protocol. The analysis results for the total AF duration and the probability of AF induction are depicted in Figure 2, B and C. There was no significant difference before surgery among the groups. However, vehicle-treated animals showed a significant increase in susceptibility to AF compared with sham rats and displayed a longer duration of AF episodes (145.81 ± 20.66 seconds versus 35.17 ± 6.34 seconds, *P* < 0.001) and a significantly increased probability of developing AF (41.70% ± 6.8% versus 86.10% ± 6.33%, *P* < 0.01) 3 days after surgery. Strikingly, treatment with GSK2193874 had a notable effect on preventing AF promotion and resulted in a shorter duration of AF episodes (41.29 ± 11.86 seconds, *P* < 0.01 versus vehicle) and a lower probability of AF (51.60% ± 10.90%, *P* < 0.05 versus vehicle).

**Figure 3:**
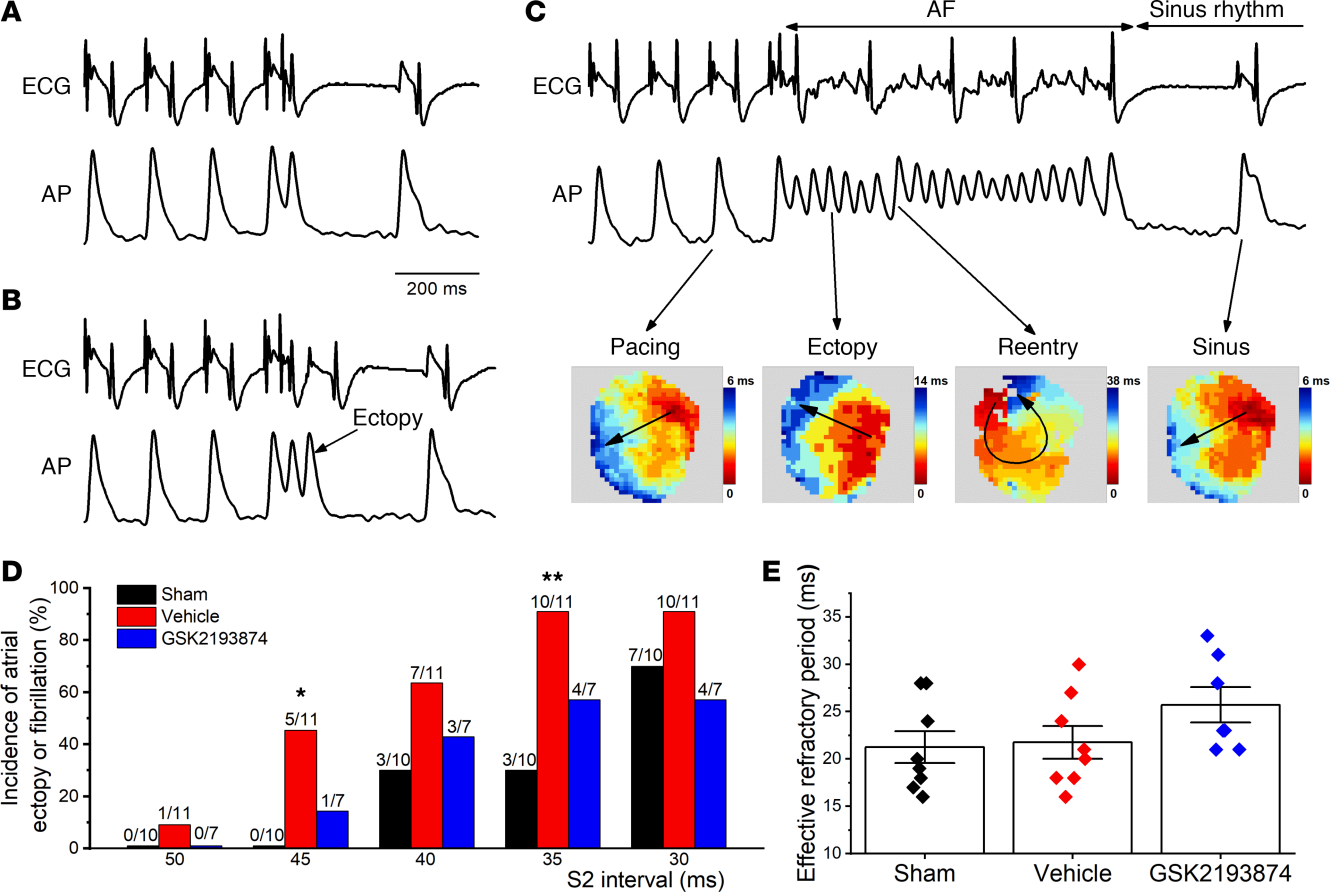
Blockage of TRPV4 suppresses atrial arrhythmia propensity in isolated hearts. (**A**–**C**) Representative optical action potential (AP) and ECG recorded in a vehicle rat, showing atrial ectopy (**B**), fibrillation (**C**), and none of both (**A**) induced by using an extrastimulus (S1S2; S2 intervals ranging from 50 to 30 ms) method. (**C**) Activation maps of pacing, ectopy, reentry, and sinus rhythm corresponding to the AP traces. (**D**) Incidence of atrial ectopy or fibrillation for each S2 interval in the 3 groups; sham, *n* = 10; vehicle, *n* = 11; GSK2193874, *n* = 7. Statistical analyses: χ^2^ test; *P < 0.05, **P < 0.01 versus sham. (**E**) Quantification of atrial effective refractory period in the 3 groups; sham *n* = 8; vehicle *n* = 8; GSK2193874 *n* = 7. Statistical analyses: 1-way ANOVA with Bonferroni’s post hoc test. Data are expressed as the mean ± SEM.

The propensity to atrial arrhythmia was also assessed in isolated hearts using optical mapping and S1S2 stimulation. Representative optical AP traces and corresponding ECG are shown in Figure 3, A–C, which illustrates a range of arrhythmic responses observed in a vehicle rat. Of note, activation map analyses of the induced AF show atrial ectopy and reentry circuit. Compared with sham rats, the hearts of vehicle-treated rats exhibited more frequent atrial ectopy or fibrillation in response to S1S2 stimulation, which was prevented after treatment with GSK2193874 (Figure 3D). However, no difference in atrial effective refractory period (AERP) was detected (Figure 3E).

### TRPV4 antagonist GSK2193874 prevents atrial electrical remodeling in SP rats.

We measured left atrial (LA) AP and conduction velocity (CV) from our optical mapping studies (Supplemental Figure 3). These data show AP duration (APD) prolongation in vehicle-treated rats, which was prevented by treatment with GSK2193874. However, no difference in time to peak or CV was detected.

**Figure 4:**
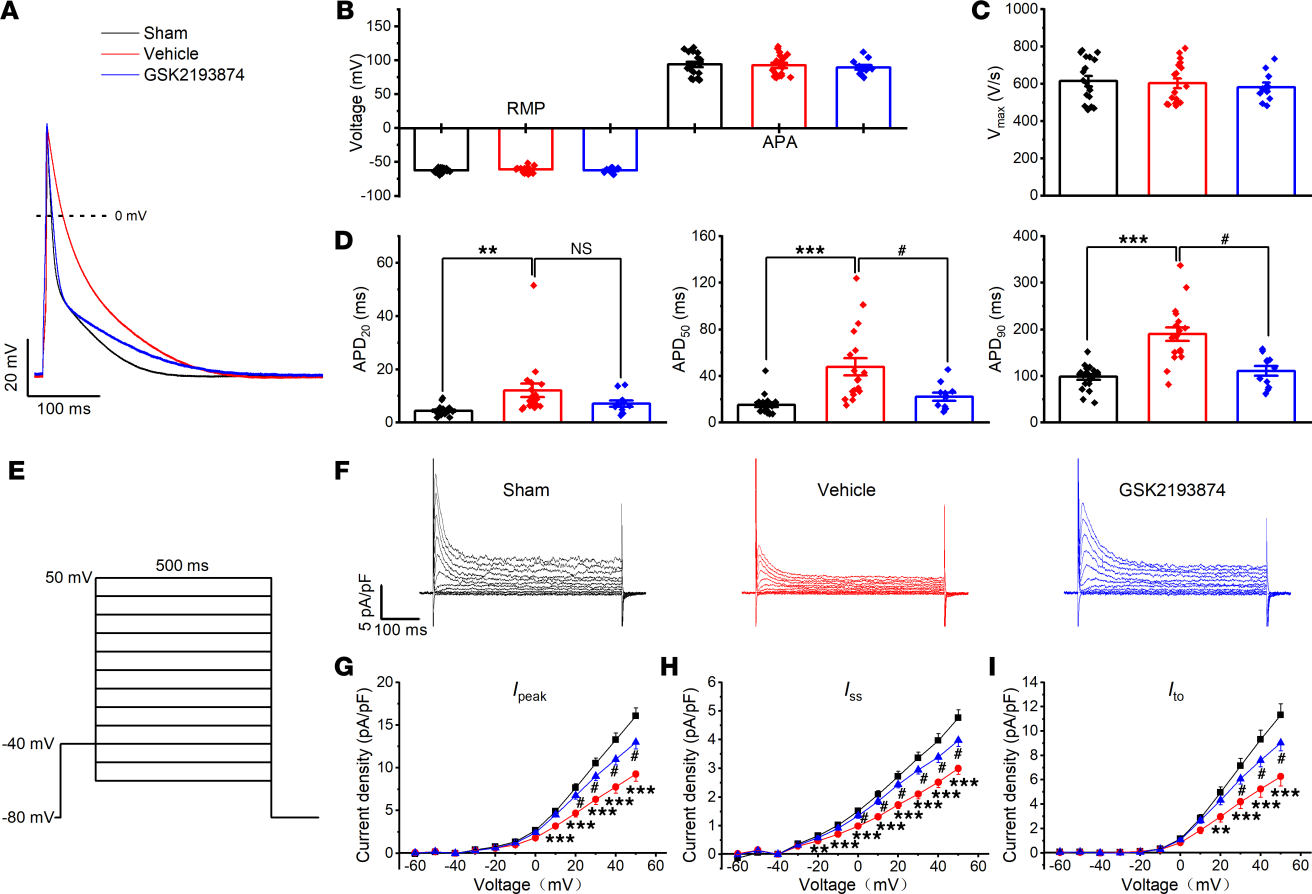
Blockage of TRPV4 prevents atrial electrical remodeling in sterile pericarditis rats. (**A**) Representative action potentials (APs) recorded from isolated atrial myocytes of indicated groups. (**B**–**D**) Mean rest membrane potential (RMP) and AP amplitude (APA) (**B**), AP slope (**C**), and action potential duration (APD) (**D**) until 20%, 50%, and 90% of repolarization (APD_20_, APD_50_, and APD_70_, respectively) in atrial myocytes. (**E**) Voltage clamp protocol. (**F**) Representative the outward voltage-gated K^+^ currents (*I*_K_) recorded from isolated atrial myocytes of indicated groups.(**G**–**I**) Mean current-voltage (I–V) curves for the peak (*I*_peak_, **G**), sustained (*I*_ss_, **H**), and transient (*I*_to_, **I**). Sham, *n* = 19 myocytes/6 rats; vehicle, *n* = 18 myocytes/7 rats; GSK2193874, *n* = 11 myocytes/7 rats. Statistical analyses: 1-way ANOVA with Bonferroni’s post hoc test (**B**, **C**, **D**, **G**, **H**, and **I**); *P < 0.05, **P < 0.01, ***P < 0.001 versus sham; ^#^*P* < 0.05 versus vehicle. Data are expressed as the mean ± SEM.

AP was also measured in atrial myocytes isolated from sham or from SP rats treated with vehicle or GSK2193874 (Figure 4, A–D). Consistent with our optical mapping findings, APD was prolonged significantly in myocytes from vehicle myocytes at 20% (APD_20_), APD_50_, and APD_90_ repolarization (*P* < 0.01 in APD_20_ and *P* < 0.001 in APD_50_, APD_90_ versus sham). This abnormal prolongation was reversed by treatment with GSK2193874 (*P* < 0.05 in APD_50_, APD_90_ versus vehicle). However, there was no significant difference in resting membrane potential (RMP), AP amplitude (APA), or maximum upstroke slope of AP (V_max_).

We further investigated the effect of TRPV4 antagonist GSK2193874 on *I*_K_, the main drivers of the cardiac repolarization. *I*_K_ was measured between –60 and +50 mV using voltage clamp protocols (Figure 4E). The representative recordings from the sham, vehicle, and GSK2193874 groups are shown in Figure 4F. Summary current-voltage (I–V) curves of the peak (*I*_peak_), sustained (*I*_ss_), and transient outward (*I*_to_) are shown in Figure 4, G–I. Vehicle treatment induced a significant reduction in *I*_peak_, *I*_ss_, and *I*_to_ (all *P* < 0.001 versus sham_,_ at +50 mV). Treating rats with GSK2193874 also prevented the reduction in *I*_peak_, *I*_ss_, and *I*_to_ (all *P* < 0.05 versus vehicle), consistent with the improvements in AP morphology. However, there were no obvious changes in the inward rectifier potassium current (*I*_K1_) and sodium current (*I*_Na_) between sham and vehicle-treated rats (Supplemental Figure 4). L-type Ca current (*I*_CaL_) was modestly decreased in the vehicle group (Supplemental Figure 5). However, the reduction in *I*_CaL_ favors shortened repolarization. We also investigated the acute effect of GSK2193874 on AP and *I*_K_ in atrial cardiomyocytes from sham and vehicle-treated rats but did not observe any significant changes (Supplemental Figure 6).

To test whether TRPV4 is functionally active in atrial myocytes, we measured Ca^2+^ using a Fluo-4 and TRPV4 agonist GSK1016790A. GSK1016790A (300 nM) induced Ca^2+^ influx in atrial myocytes (Supplemental Figure 7A), which suggests that atrial myocytes express active TRPV4 channels. We further investigated the acute effect of GSK1016790A on AP and *I*_K_ in atrial cardiomyocytes from vehicle-treated rats. As shown in Supplemental Figure 7B, APD was prolonged ~5 minutes after the application of GSK1016790A. However, nearly all of the atrial myocytes died before APD reach stability. As described previously (11), the upregulation of TRPV4 can further increase AP-induced Ca^2+^ transients, which were mediated via increased SR Ca^2+^ content and the facilitation of ryanodine receptor Ca^2+^ release. Thus, myocytes may die from the increased cellular Ca^2+^ stress induced by GSK1016790A. Meanwhile, the acute application of GSK1016790A did not affect *I*_K_ in atrial myocytes (Supplemental Figure 7C). Note that *I*_K_ was measured at a testing potential of +50 mV from a holding potential of –80 mV (Supplemental Figure 7C). Under this circumstance, *I_CaL_* (only ~20% of peak current) was low (Supplemental Figure 5), and ryanodine receptor Ca^2+^ release could hardly be induced. Therefore, the cells remained alive even in the presence of GSK1016790A.

### TRPV4 antagonist GSK2193874 reduces interstitial fibrosis in SP rats.

**Figure 5:**
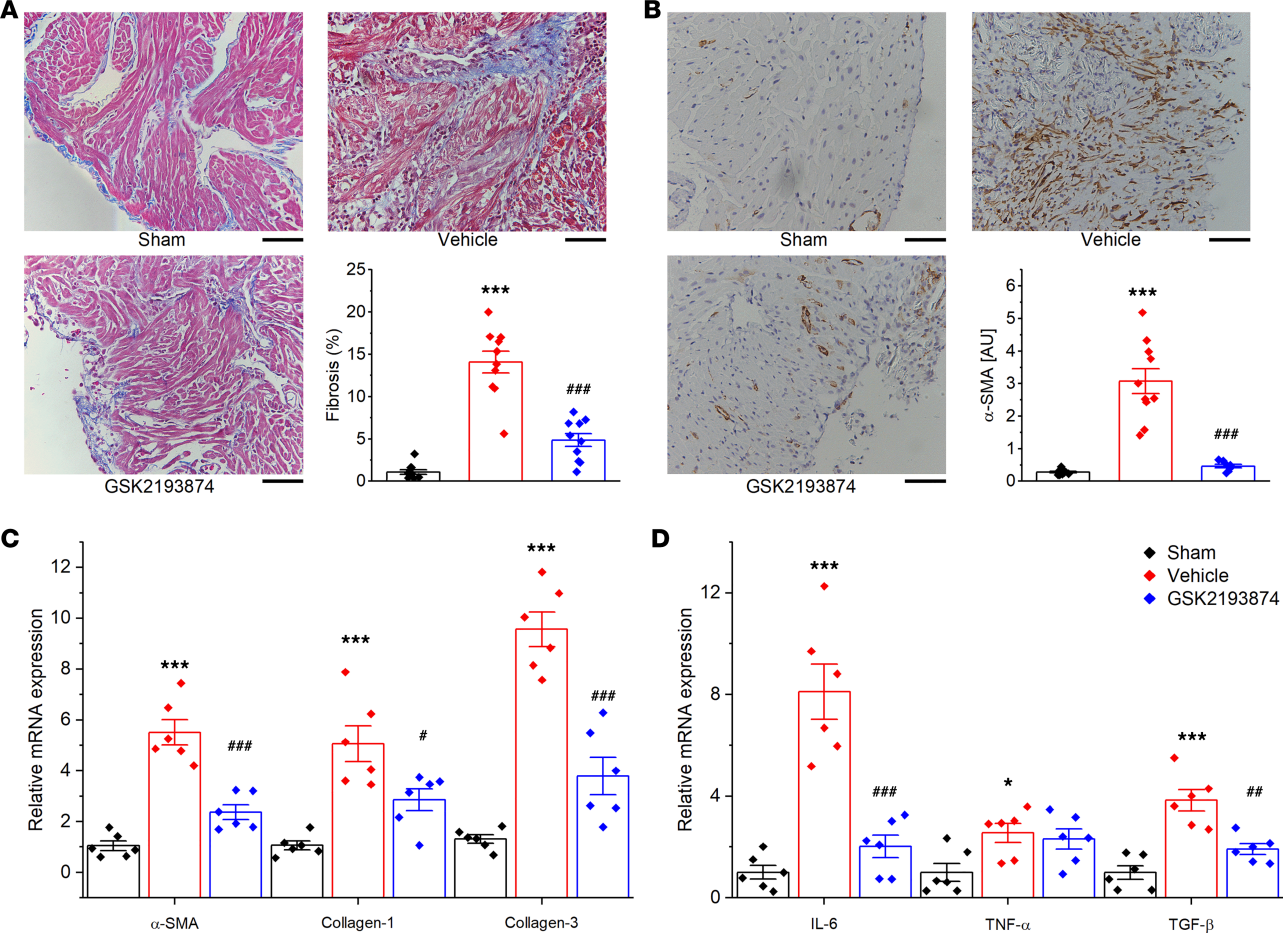
Blockage of TRPV4 attenuates atrial fibrosis and related gene expression in sterile pericarditis rats. (**A**) Representative histological sections stained with Masson trichrome and percentage of left atrial interstitial fibrosis. *n* = 10/group. Scale bars: 50 μm.(**B**) Examples of α-SMA immunohistochemical staining and quantification. Sham, *n* = 10; vehicle, *n* = 10; GSK2193874, *n* = 8. Scale bars: 50 μm. (**C**) The mRNA expression of α-SMA, collagen-1, and collagen-3 by real-time PCR. *n* = 6/group, each in triplicate. (**D**) The mRNA expression of IL-6, TNF-α, and TGF-β by real-time PCR. *n* = 6/group, each in triplicate. Statistical analyses: 1-way ANOVA with Bonferroni’s post hoc test (**A**–**D**); *P < 0.05, ***P < 0.001 versus sham; ^#^*P* < 0.05, ^##^*P* < 0.01, ^###^*P* < 0.001 versus vehicle. Data are expressed as the mean ± SEM.

Interstitial fibrosis was evaluated on day 3 by Masson’s trichrome staining of atria. Figure 5A shows representative images and quantitative results. Vehicle-treated rats displayed distinct atrial fibrosis (*P* < 0.001 versus sham). GSK2193874 treatment markedly reduced the amount of atrial fibrosis (*P* < 0.001 versus vehicle). Immunostaining showed that the expression of α-SMA in the atria increased in the vehicle-treated rats (*P* < 0.001 versus sham, Figure 5B). GSK2193874 reduced the expression of α-SMA (*P* < 0.001 versus vehicle). Next, we examined the expression of molecules related to profibrotic and proinflammatory signaling. Expressions of collagen-1, collagen-3, α-SMA, IL-6, TNF-α, and TGF-β1 were significantly upregulated in the atria of vehicle-treated rats compared with sham rats. Again, GSK2193874 treatment attenuated all the effects above (Figure 5, C and D). We previously found that the infiltration of immune cells was increased significantly in the atria from SP rats (19). Here, we observed the number of MPO^+^ cells (neutrophil) and CD68^+^ cells (macrophages) was reduced after GSK2193874 treatment (*P* < 0.01 in MPO^+^ and *P* < 0.001 in CD68^+^ versus vehicle; Supplemental Figure 8).

### TRPV4 antagonist GSK2193874 inhibits the activation of P38, AKT, and STAT3 in SP rats.

**Figure 6:**
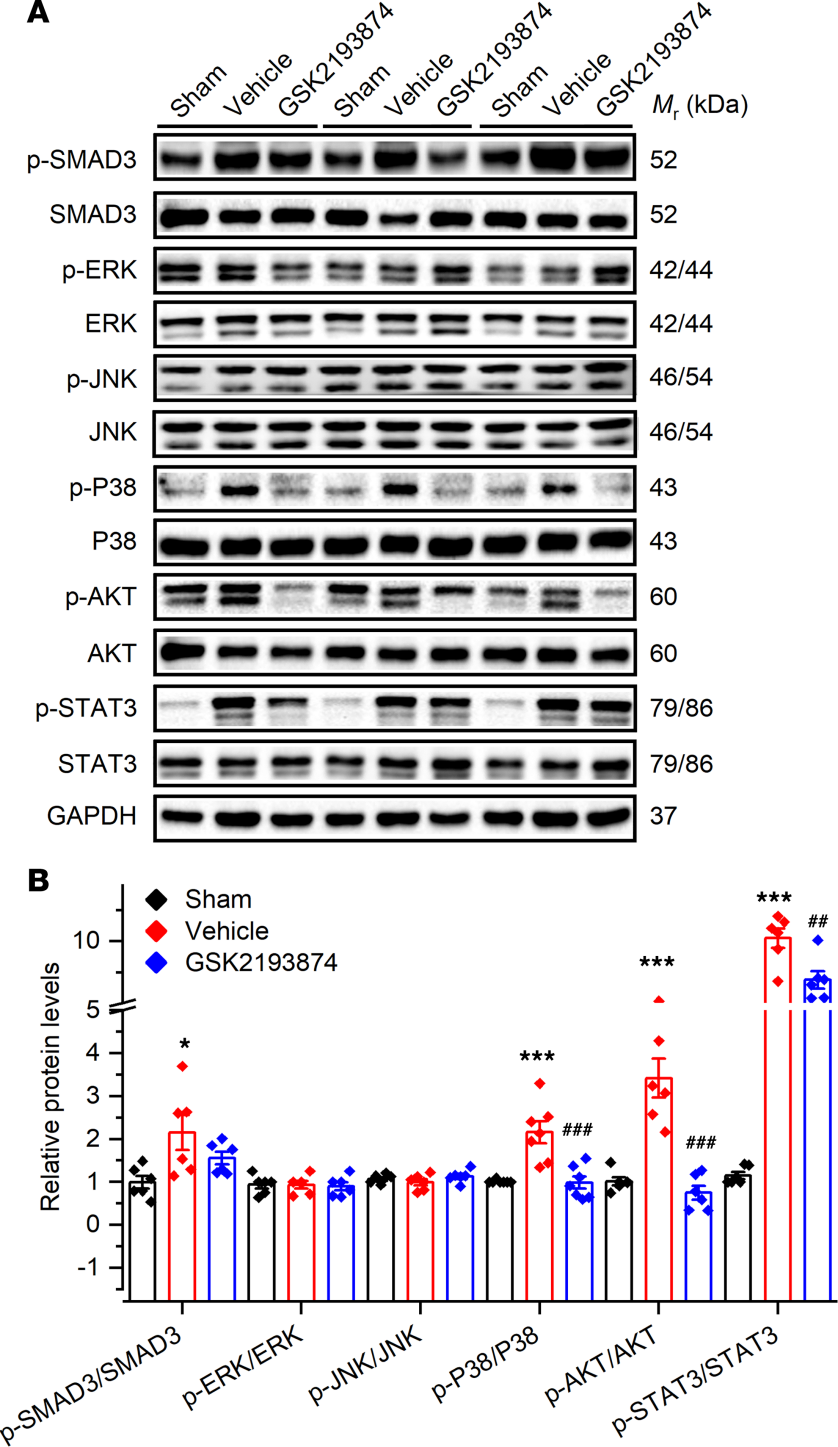
Effect of TRPV4 blockage on atrial fibrosis-related signaling pathways in sterile pericarditis rats. (**A** and **B**) Representative Western blot (**A**) and quantification (**B**) of SMAD3, p-SMAD3, ERK, p-ERK, P38, p-P38, JNK, p-JNK, AKT, p-AKT, STAT3, and p-STAT3 in atrial tissue of indicated group. *n* = 6–7/group. Statistical analyses: 1-way ANOVA with Bonferroni’s post hoc test (**B**); *P < 0.05, ***P < 0.001 versus sham; ^##^*P* < 0.01, ^###^*P* < 0.001 versus vehicle. Data are expressed as the mean ± SEM.

We then investigated the molecular mechanism by which GSK2193874 treatment reduced interstitial fibrosis in SP rats. SMAD, ERK, P38, JNK, AKT, and STAT3 are downstream effectors of atrial profibrotic mediator TGF-β1 and play a key role in atrial fibrosis (6, 20, 21). We measured the degree of activation of SMAD3, ERK, P38, JNK, AKT, and STAT3 using antibodies against phosphorylated SMAD3 (p-SMAD3), p-ERK, p-P38, p-JNK, p-AKT, or p-STAT3 on day 3. Figure 6 shows typical Western blot results and the quantitative results, and it demonstrates that the phosphorylation of SMAD3, P38, AKT, and STAT3 was more pronounced in vehicle-treated rats than in sham rats. As predicted, the degree of P38, AKT, and STAT3 phosphorylation decreased in the TRPV4 antagonist GSK2193874 treatment rats, whereas SMAD3 phosphorylation was not affected. In accordance with one of our previous studies, the relative level of phosphorylated ERK1/2 did not change on day 3 after pericardiotomy and was not significantly different among groups (6). The activation of JNK was not significantly different among groups, either.

### TRPV4 is involved in TGF-β1–induced atrial fibroblasts differentiation.

Previous work demonstrated that TRPV4 is functionally expressed in ventricular fibroblasts and plays an essential role in regulating differentiation (15). To investigate the role of TRPV4 in atrial fibroblast differentiation, we examined the mRNA expression of TRPV4 in cultured atrial fibroblasts with or without TGF-β1 by real-time PCR. Stimulation of atrial fibroblasts with TGF-β1 (10 ng/mL) for 24 hours produced a significant increase in TRPV4 mRNA expression (Supplemental Figure 9A). Similarly, Ca^2+^ influx induced by the TRPV4 agonist GSK1016790A (300 nM) was significantly enhanced after stimulation with TGF-β1 (Supplemental Figure 9, B and C). In line with previous research, TGF-β1 treatment induced robust differentiation of atrial fibroblasts to myofibroblasts, as evidenced by an increase in the mRNA expression of α-SMA, collagen-1, and collagen-3 (15, 22). Importantly, pretreatment with the TRPV4 antagonist GSK2193874 significantly inhibited TGF-β1–induced differentiation of atrial fibroblasts. In contrast, TRPV4 agonist GSK1016790A further enhanced TGF-β1–induced atrial fibroblast differentiation (Supplemental Figure 9D).

### The function of TRPV4 is enhanced in the atrial fibroblasts from SP rats.

**Figure 7:**
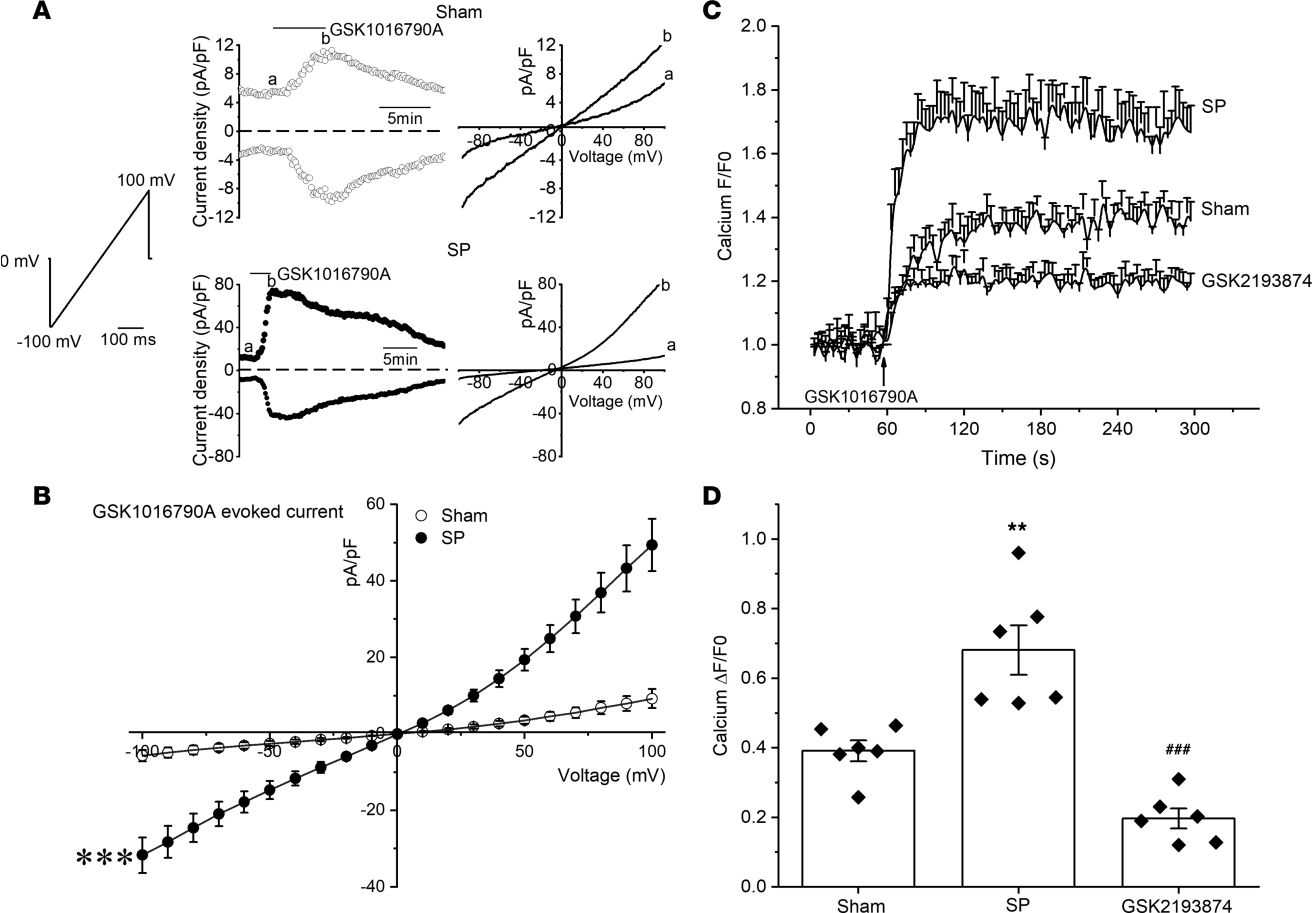
The function of TRPV4 enhances in atrial fibroblasts from sterile pericarditis rats. (**A**) Time course of whole-cell current at +90 and –90 mV evoked by 300 nM GSK1016790A (left panel) and current-voltage (I–V) relations taken at time points a and b (right panel) in atrial fibroblasts from sham and SP rats. A ramp protocol elicited by a voltage ramp from –100 mV to +100 mV. Horizontal bars denote the time courses for applications of GSK1016790A. (**B**) Mean current-voltage (I–V) curves for GSK1016790A-induced TRPV4 current. Sham, *n* = 8 cells/5 rats; SP, *n* = 9 cells/5 rats. (**C** and **D**) Representative time course (**C**) of the changes in [Ca^2+^]_i_ and quantification (**D**) induced by GSK1016790A in atrial fibroblasts from sham and cells from SP rats with/without pretreatment with a selective TRPV4 antagonist, GSK2193874 (300 nM). *n* = 6/group. Statistical analyses: 2-tailed unpaired Student’s *t* test (**B**) and 1-way ANOVA with Bonferroni’s post hoc test (**D**); **P < 0.01, ***P < 0.001 versus sham; ^###^*P* < 0.001 versus SP; **P < 0.01 versus sham; ^###^*P* < 0.001 versus SP. Data are expressed as the mean ± SEM.

To determine whether its function is enhanced in atrial fibroblasts from SP rats, we performed patch clamp and intracellular Ca^2+^ measurements in freshly dissociated atrial fibroblasts. Cells were voltage clamped at a holding potential of 0 mV, and voltage ramps (± 100 mV over 400 ms) were applied every 10 seconds. TRPV4 agonist GSK1016790A (300 nM) induced inward and outward transmembrane currents at –90 mV and +90 mV, respectively (Figure 7A). Figure 7B shows the average GSK1016790A-evoked currents in atrial fibroblasts from sham and SP rats. Atrial fibroblasts from SP rats displayed an increased current density compared with the sham group (*P* < 0.001). Consistent with the electrophysiological experiments, Ca^2+^ influx induced by 300 nM GSK1016790A significantly increased in atrial fibroblasts from SP rats, which was markedly reduced by the TRPV4 antagonist GSK2193874 (Figure 7C). Figure 7D shows the quantitative analysis of relative changes (ΔF/F0) in Ca^2+^ influx at the steady state.

### TRPV4 contributes to the differentiation and proliferation of atrial fibroblasts from SP rats via the activation of P38, AKT, and STAT3.

**Figure 8:**
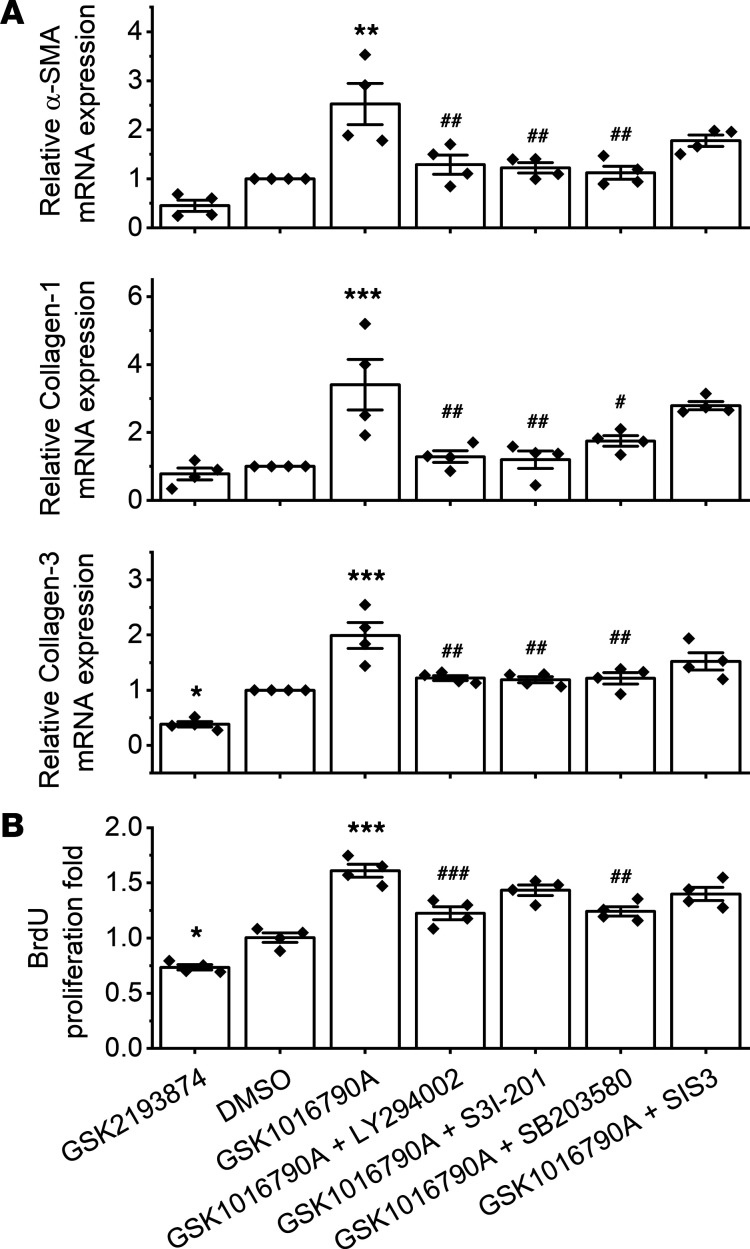
TRPV4 contributes to the differentiation and proliferation of atrial fibroblasts from sterile pericarditis rats via the activation of P38, AKT, and STAT3. (**A**) The mRNA expression of α-SMA, collagen-1, and collagen-3 by real-time polymerase chain reaction (PCR). *n* = 4/group. (**B**) Proliferation of CFs by BrdU assay. Cells were treated with DMSO or GSK1016790A, or with GSK2193874 or GSK2193874 + multiple signaling pathway inhibitors. LY294002, an AKT inhibitor; S3I-201, a STAT3 specific inhibitor; SB 203580, P38 inhibitor; or SIS3, a SMAD3 inhibitor. *n* = 4/group, each in triplicate. Statistical analyses: 1-way ANOVA with Bonferroni’s post hoc test (**D**); *P < 0.05, **P < 0.01, ***P < 0.001 versus DMSO; ^#^*P* < 0.05, ^##^*P* < 0.01, ^###^*P* < 0.001 versus GSK1016790A. Data are expressed as the mean ± SEM.

To further investigate whether TRPV4 might be involved in the activation of atrial fibroblasts from SP, we measured the fibrosis-related genes collagen-1, collagen-3, α-SMA by real-time PCR (Figure 8A), and we measured cell proliferation by BrdU (Figure 8B). The fibrosis-related genes and cell proliferation were significantly reduced by treatment with TRPV4 antagonist GSK2193874 but were markedly enhanced by TRPV4 agonist GSK1016790A. Moreover, GSK1016790A-induced activation of atrial fibroblasts from SP was blunted by LY294002 (an AKT antagonist), S3I-201 (a STAT3 antagonist), or SB 203580 (a P38 inhibitor). However, pretreatment with SMAD3 inhibitor SIS3 had no effect.

## Discussion

We recently demonstrated that SP rats show that a higher incidence of AF is associated with the disruption of conduction homogeneity, as well as increases in atrial fibrosis (6). In the current study, we found that SP rats also display substantial alterations in atrial electrophysiology, including abnormal prolongation of APD and downregulation of *I*_K_. Furthermore, ex vivo optical mapping revealed increased atrial ectopy and AF with a reentrant pattern. Prolonged atrial APD could increase the likelihood of ectopy firing, and atrial fibrosis could stabilize reentry. These might be the mechanisms through which AF is promoted in SP rats. Strikingly, in the present study, we discovered that the expression of TRPV4 increased in the atria of SP rats and that the blockade of TRPV4 attenuated SP-induced atrial profibrillatory remodeling. These results suggest that TRPV4 can be a potentially novel target to prevent postoperative AF.

TRPV4 is widely expressed in most mammalian cells (23). Its expression in the heart is generally lower under basal conditions but can increase following ischemia-reperfusion and pressure overload, which commonly occur during cardiac surgery (9, 11, 24). TRPV4 activation contributes to cardiomyocyte apoptosis, myofibroblast differentiation, fibrosis, adverse remodeling, and cardiac dysfunction (10, 15, 25, 26). Elevated mRNA expression of leukocyte TRP channels, including TRPV4, has been found in patients with nonvalvular AF (27). Our data show that the protein expression of TRPV4 increased in the atria of SP rats, as well as in AF patients. However, unlike the mechanisms in SP rats, the increase in TRPV4 expression in AF patients may be due to presurgery factors. Nevertheless, our data indicate that the upregulation of TRPV4 in both atrial myocytes and fibroblasts may contribute to AF.

Our results show that treatment with TRPV4 antagonist GSK2193874 prevented inducible AF and ameliorated atrial electrical and structural remodeling in SP rats. In terms of electrical remodeling, we observed that SP rats exhibited abnormal prolongations in APD. APD prolongation was also observed in patients with lone paroxysmal AF, in the atrial tissue of patients predisposed to AF, and in various patient and animal studies of AF with underlying structural changes in the atria (28, 29). However, in human AF, particularly persistent AF, APD was reported to have shortened or remained unchanged. The downregulation of repolarizing *I*_K_ can significantly alter AP shape and duration, contributing to electric remodeling (30). A marked decrease in *I*_K_ was previously documented in both animal models and patients with AF. *I*_K_ reduction and atrial AP prolongation could increase the likelihood of early-after depolarization, which could contribute to increased AF susceptibility in SP rats (30). Treatment with GSK2193874 prevented APD prolongations and *I*_K_ reduction, suggesting that treatment with GSK2193874 may reduce the atrial electric remodeling by the upregulation of *I*_K_. In addition, we found that RMP, APA, and V_max_ were not altered in SP rats, which was consistent with the findings that *I*_K1_ and *I*_Na_ were not modulated in SP rats.

TGF-β1 has been identified as a major driving force for fibroblast to myofibroblast differentiation in vitro and tissue fibrosis of many organs (20). Similar to previous studies in ventricular fibroblasts, TRPV4 functional expression significantly increased when atrial fibroblasts were exposed to TGF-β1 (15). Importantly, TGF-β1–induced fibroblast differentiation was attenuated by the TRPV4 antagonist GSK2193874 but was enhanced by the TRPV4 agonist GSK1016790A. Our findings also suggest that TRPV4 plays a critical role in atrial fibrosis in vivo in SP rats by regulating fibroblast differentiation. Indeed, atrial fibroblasts isolated from SP rats exhibited higher TRPV4 activity. Both current density and Ca^2+^ influx mediated by TRPV4 from SP rats were significantly larger than those from sham animals. However, Du et al. reported no detected TRPV4 currents in isolated human atrial fibroblasts, even with abundant expression at the mRNA level (31). Using patch clamp and Ca^2+^ imaging techniques, we detected TRPV4 currents and TRPV4-mediated Ca^2+^ influx in rat atrial fibroblasts. This difference may be related to the different in species or TRPV4 agonists. The present study chose a more potent agonist (GSK1016790A versus 4α-PDD) (32). Nevertheless, our results indicate that the activation of TRPV4 contributed to atrial fibrosis and TGF-β1–induced atrial fibroblast differentiation.

TRPV4 has been detected in immune cells (like monocytes, macrophages, and neutrophils), and its activation promotes the infiltration of immune cells and the release of inflammatory cytokines (33). We also found that treatment with GSK2193874 reduced the infiltration of immune cells and the expression of IL-6 and TGF-β, suggesting that the inhibition of immune cell infiltration may also contribute to the therapeutic effects of TPRV4 blockade in SP rats. To gain a better understanding of the precise mechanism of the hypothesis would further investigation.

We found that the activation of P38, AKT, and STAT3 played an important role in TRPV4-mediated atrial fibrosis. Of note, treatment with TRPV4 antagonist GSK2193874 had no significant effect on the activation of SMAD3, the canonical TGF-β1 signaling pathway (34). This result suggests that TRPV4 mediates fibrosis via an SMAD3-independent pathway, which is consistent with previous research (35). Furthermore, we found that SMAD inhibitor SIS3 had no effect on the differentiation and proliferation of isolated atrial fibroblasts from SP rats, indicating that SMAD3 does not regulate the activation of atrial fibroblasts mediated by TRPV4 in SP rats. However, the activation of atrial fibroblasts was significantly attenuated by P38 inhibitor SB 203580, AKT inhibitor LY294002, and STAT3 inhibitor S3I-201. In addition, treatment with GSK2193874 reduced atrial fibrosis and prevented SP-induced collagen1, collagen3, α-SMA, IL-6, and TGF-β expression in vivo. Meanwhile, the activation of P38, AKT, and STAT3 was significantly inhibited. These findings suggest that TRPV4 blockade reduces atrial fibrosis and the activation of atrial fibroblasts by inhibiting the P38, AKT, and STAT3 pathways in SP rats.

In general, the mechanisms leading to AF are underexamined but are thought to involve ectopic (triggered) activity in the setting of a substrate that favors reentry (1). No changes in AERP and CV were observed in SP rats, which means that the likelihood of reentry should not be increased. However, prolonged APD, while favoring triggered activity, might also increase the dispersion of repolarization and favor reentry. Furthermore, atrial fibrosis interferes with local conduction and promotes the maintenance of AF. Treatment with TRPV4 antagonist GSK2193874 ameliorated arrhythmogenic substrate in the atrium by reversing abnormal APD prolongation, *I*_K_ reduction, and atrial fibrosis. However, acute application of GSK2193874 did not affect AP or *I*_K_ in atrial cardiomyocytes from sham or SP rats, implying that the attenuation of atrial fibrosis is the predominant mechanism of GSK2193874. Moreover, the acute application of TRPV4 agonist GSK1016790A (~5 minutes) produced an increase in APD but had no effect on *I*_K_. These findings suggest that TRPV4 activation contributes to prolonged APD, which could be at least partially reversed after treatment with GSK2193874. However, nearly all of the atrial myocytes died before APD reached stability after being perfused with GSK1016790A, and further research with more advanced techniques is needed to clarify the reasons. In addition, TGF-β1 has been found to affect the electrophysiology properties of cardiomyocytes by altering the cross-talk between cardiomyocytes and fibroblasts (36). Therefore, treatment effects of GSK2193874 on APD and *I*_K_ in atrial cardiomyocytes might be through the blockade TGF-β1 signaling, which requires further research.

### Study limitations.

Some limitations should be acknowledged when considering the results of the present study. First, although the upregulation of TRPV4 was consistent in SP rats (in vivo and in vitro atrial fibroblasts) and in the atrial tissue from AF patients, our data do not provide conclusive evidence about the involvement of TRPV4 in AF progression in patients. Further human studies are needed to verify our results. Second, we only used pharmacological tools to modulate TRPV4 and to examine its effect on atrial remodeling. However, all pharmacological probes, especially ion channel blockers, are imperfectly specific, and we cannot exclude the possibility of off-target effects. Third, although we proved that TRPV4 blockade prevented the induction and maintenance of AF in SP rats, these results could not be extrapolated to other animal AF models or non-postoperative AF patients. Fourth, we only assessed the role of TRPV4 in atrial electrical and structural remodeling; additional studies focusing on abnormal intracellular Ca^2+^ dynamics, neurohormonal dysregulation, and the mechanisms of TRPV4 activation are also warranted.

### Conclusion.

The present study provides the first evidence to our knowledge that TRPV4 expression is increased in the atrial tissues from SP rats and patients with AF. The blockade of TRPV4 reduced AF and atrial remodeling in SP rats. In terms of the mechanisms, TRPV4 antagonist GSK2193874 reversed the abnormal APD prolongation in atrial myocytes through the regulation of *I*_K_; reduced the activation of atrial fibroblasts by inhibiting P38, AKT, and STAT3 pathways; and alleviated the infiltration of immune cells. Thus, TRPV4 may be critical to the development of AF, particularly postoperative AF, and may be a potential target for therapeutic intervention. More research using other animal models is needed to confirm our findings regarding the impact of TRPV4 on AF and to evaluate the potential of TRPV4 antagonists as a therapeutic strategy for AF.

## Methods

### Animal model and treatment.

Male Sprague-Dawley rats weighing 180 to 250 g were purchased from the Experimental Animal Center, Tongji Medical College. All rats were allowed free access to food and water before the operation under optimal conditions (12 hours light/12 hours dark with humidity 60%, 24°C).

Under general anesthesia (i.p. sodium pentobarbital, 40 mg/kg), pericardiotomy was performed to dust with sterile talcum powder on both atria as previously described (5). The SP rats were treated with vehicle (6% Cavitron) or TRPV4 antagonist GSK2193874 (10 mg/kg/day, Sigma-Aldrich) via oral gavage for 3 days. Treatment started 1 day before pericardiotomy. The concentration of GSK2193874 was selected based on previous studies (18). The sham-operated animals were subjected to the same procedure without pericardiotomy. Instrumentation and AF induction were performed on the third postoperative day, as previously described (6). AF was defined as a rapid and irregular atrial rhythm (fibrillatory baseline in the ECG) with irregular RR intervals lasting at least 2 seconds on the ECG. The probability of AF induction and total AF duration was analyzed. At the end of the experiments, atria were immediately washed in phosphate-buffered saline (PBS), immersed into 4% paraformaldehyde or flash-frozen with liquid nitrogen, and stored at –80°C until subsequent examination.

### Human tissue sample.

Right atrial appendage biopsy samples were obtained from patients in sinus rhythm and with chronic AF during coronary artery bypass graft surgery (patient clinical information is shown in Supplemental Table 1). Right atrial tissue samples were collected and fast-frozen in liquid nitrogen.

### Epicardial activation mapping.

On day 3 after surgery, the rats were euthanized (pentobarbital sodium; 40 mg/kg) and heparinized (120 IU). Hearts were excised and retrogradely perfused via the aorta with Tyrode solution (in mM: 128.2 NaCl, 4.7 KCl, 1.3 CaCl_2_, 1.05 MgCl_2_, 1.19 NaH_2_PO_4_, 20 NaHCO_3_, and 11.1 glucose; 95% O_2_/5% CO_2_) at 10 mL/min and 37°C. RH237 (Invitrogen) was used to index membrane potential changes (0.05 mg in 50 mL Tyrode solution for 5 minutes). Blebbistatin (Abcam, 10 μM) was used as an uncoupler to avoid motion artifacts. To record optical AP, the left atria was illuminated with light-emitting diodes (LEDC-2001, MappingLab) at a wavelength of 530 nm. The emitted fluorescence signal of transmembrane potential (Vm) was long-passed (>700 nm) and acquired via a CMOS camera (OMS-PCIE-2002, MappingLab). Digital images (64 × 64 pixels) were gathered at a sampling rate of 0.5 kHz from a 6.4 × 6.4 mm field of view. An ECG was continuously recorded. A bipolar lead was used to pace the right atrium. Baseline electrophysiological parameters were measured at a pacing rate of 7 Hz. AERP and arrhythmia propensity were determined using an extrastimulus (S1S2; 30 S1 stimuli at 7 Hz followed by a premature S2 stimulus ranging from 50 to 15 ms) protocol.

Optical mapping data were analyzed using a commercially available software (OMapScope4.0, MappingLab). Optical signals were spatially aligned and processed using a Gaussian spatial filter (3 × 3 pixels). To improve signal quality, 64 × 64 pixels were transformed to 32 × 32 pixels when the atrial fibrillation signals were analyzed. Local activation time was assigned based on the maximum departure velocity of the Vm upstroke. Time to peak was defined as the time from initiation of AP to peak fluorescence. APD_20_, APD_50_, and APD_80_ were calculated. CV was determined by the space-time coordinates of local activation.

### Western blot analysis.

Western blot was performed as described previously (9). Samples (20 μg) were run on a 10% SDS-PAGE gel followed by blotting to a nitrocellulose membrane. Membranes were blocked and incubated with the following antibodies: TRPV4 (ACC-034, Alomone Labs), SMAD3 (9523, Cell Signaling Technology), p-SMAD 3 (9520, Cell Signaling Technology), ERK (4695, Cell Signaling Technology), p-ERK (4370, Cell Signaling Technology), JNK (9252s, Cell Signaling Technology), p-JNK (4671s, Cell Signaling Technology), P38 (9212s, Cell Signaling Technology), p-P38 (4511s, Cell Signaling Technology), AKT (4691,Cell Signaling Technology), p-AKT (4060, Cell Signaling Technology), STAT3 (9132, Cell Signaling Technology), and p-STAT3 (9131, Cell Signaling Technology). Corresponding secondary antibodies conjugated to horseradish peroxidase were used for detection. Staining was detected using chemiluminescence and quantified by Image Lab software (Bio-Rad). All expression data was provided relative to GAPDH (diluted 1:500, Aspen) staining for the same samples on the same gels.

### Real-time PCR.

Total RNA was isolated from atrial tissues or cultured atrial fibroblasts using TRIzol reagent (Takara Bio). Gene-specific primers used in the study were: IL-1β, 5′-CTCTGTGACTCGTGGGATGATG-3′ (forward), 5′-CACTTGTTGGCTTATGTTCTGTCC-3′ (reverse); IL-6, 5′-AACGAAAGTCAACTCCATCTG-3′ (forward), 5′-GGTATCCTCTGT GAAGTCTCC-3′ (reverse); TGF-β1, 5′-TGGCGTTACCTTGGTAACC-3′ (forward), 5′-GGTGTTGAGCCCTTTCCAG-3′ (reverse); TNF-α, 5′-CCCAGACCCTCACACTCAGATC AT-3′ (forward), 5′-GCAGCCTTGTCCCTTGAAGAGAA-3′ (reverse); collagen-1, 5′-GA GCGGAGAGTACTGGATCG-3′ (forward), 5′-TACTCGAACTGGAATCCATC-3′ (reverse); collagen-3, 5′-CAGCTGGCCTTCCTCAGACT-3′ (forward), 5′-TGCTGTTTTTGCAC TGGTATGTAA-3′ (reverse); α-SMA, 5′-CTGTGCTATGTCGCTCTGGA-3′ (forward), 5′- ATAGGTGGTTTCGTGGATGC-3′ (reverse); and GAPDH, 5′-GACATCAAGAAGGTGGTGAAGC-3′ (forward), 5′-TGTCATTGAGAGCAATGCCAGC-3′ (reverse). The real-time PCR Kit (Takara) was used, and the reactions were performed in the Stepone ABI system. GAPDH was included as an internal reference. The relative expression quantity 2^(−ΔCt)^ was used to calculate the differences among groups (37).

### Atrial histology and IHC staining.

Tissue samples from the atria were fixed with 4% paraformaldehyde and embedded in paraffin. Tissues were cut into 4 μm sections and subsequently stained with Masson’s trichrome stain to evaluate LA fibrosis. Images were acquired and digitized on an Olympus BX-51 epifluorescence microscope (Olympus) with an attached digital camera, and fibrotic and normal myocardial tissue areas were analyzed at 400× magnification using Image-Pro 6.2 software. The percentage of fibrosis was determined by calculating the ratio of areas of fibrotic to normal myocardial tissue. Four images per atrium were analyzed from 6 animals per group to obtain mean values.

A separate group of sections was immunostained with primary antibodies against TRPV4 (diluted 1:50, ACC-034, Alomone Labs), MPO (diluted 1:50; ab208670, Abcam), CD68 (diluted 1:50; BA3638, Boster), or α-SMA (diluted 1:50, BM0002, Boster), followed by an incubation with biotin-conjugated secondary antibodies, and then treated with avidin-peroxidase. The reaction was developed using the DAB substrate kit (BioSci), and the sections were counterstained with H&E.

Double immunofluorescent staining was used to investigate the atrial tissues and isolated atrial myocytes/fibroblasts with the TRPV4 antibody (diluted 1:50, ACC-034, Alomone Labs), Cardiac troponin T antibody (diluted 1:100; ab8295, Abcam), and vimentin antibody (diluted 1:150; CY5134, Abway).

### Atrial myocytes isolation.

After the excision of the heart from male Sprague-Dawley rats, single atrial myocytes were isolated, as described previously, but with minor modifications (38). In short, hearts were mounted on a Langendorff perfusion apparatus and retrogradely perfused at 37°C through the aorta. The remaining blood in the hearts was washed away with an isolation solution and 750 μM Ca^2+^ (2 minutes). The solution was then changed to Ca^2+^ free isolation solution with 100 μM EGTA for 4 minutes before the solution was supplemented with enzymes containing 1 mg/mL collagenase (Worthington, Type II), 0.1 mg/mL protease (MilliporeSigma, Type XIV), and 50 μM Ca^2+^. After 6–8 minutes, the hearts were removed from the Langendorff apparatus. The atria were separated, minced into small chunks, and further digested in enzyme solution at 37°C until single myocytes with clear cross striations appeared in the suspension. Isolation solution contained (in mM) 130 NaCl, 5.4 KCl, 1.4 MgCl_2_, 0.4 NaH_2_PO_4_, 5 HEPES, 10 glucose, 20 taurine, and 10 creatine (pH 7.3, adjusted with NaOH).

For intracellular Ca^2+^ measurement, atrial myocytes were collected and purified by gravity setting, according to established procedures (39). Briefly, cell suspensions were filtered through a 100 μm pore-size strainer, and atrial myocytes were purified simply by 3 rounds of sequential gravity settling for 10 minutes.

In another experiment, the filtered cells were plated on tissue culture surfaces coated with laminin and, after 4 hours, fixed in 4% formaldehyde to analyze their expression of TRPV4 by immunofluorescence assay.

### Atrial fibroblasts isolation and treatment.

Atrial fibroblasts were isolated from the atrial tissues of sham and SP rats, as described previously (6). Briefly, tissues were digested with 100 U/mL collagenase (Type II) and 0.1% trypsin (Amresco) for 8 consecutive 7- to 10-minute treatment periods at 37°C. Atrial fibroblasts were pelleted at 1000 rpm for 10 minutes and resuspended in DMEM (Thermo Fisher Scientific) supplemented with 10% FBS (Thermo Fisher Scientific), 100 U/mL penicillin (Servicebio), and 100 μg/mL streptomycin (Servicebio). Experiments were performed with cells from the first and second passages. Before cells were treated with different chemical interventions, the FBS in the medium was deprived for 24 hours to induce growth arrest. The cells were then treated with TRPV4 antagonist GSK2193874 (300 nM) or TRPV4 agonist GSK1016790A (300 nM, Sigma-Aldrich) for an additional 24 hours in DMEM with 10% FBS in the presence of TGF-β1 (10 ng/mL, PeproTech). In another group of experiments, cells were preincubated with AKT specific inhibitor LY294002 (40 μM, Selleckchem), STAT3 specific inhibitor S3I-201 (50 μM, Selleckchem), P38 inhibitor SB 203580 (10 μM, Selleckchem), or SMAD3 inhibitor SIS3 (3 μM, Selleckchem) for 30 minutes before GSK1016790A treatment.

### Patch clamp recordings.

Whole-cell patch clamping was applied for AP and current recoding using Multiclamp 700A amplifiers (Molecular Devices), as described in our previous studies (40, 41). Electrical signals were digitized using 250 kHz 16-bit resolution A/D converters (Digidata 1322; Molecular Devices) and recorded by pCLAMP 9 software (Molecular Devices) with low-pass filtering at 2 kHz. Pipettes were pulled using a horizontal glass microelectrode puller (P-97; Sutter Instrument Co). Experiments were performed at room temperature within 6 hours after enzymatic dissociation.

For AP recording, atrial myocytes were bathed with Tyrode solution containing (in mM) 136 NaCl, 5.4 KCl, 1.8 CaCl_2_, 1 MgCl_2_, 0.33 NaH_2_PO_4_, 10 HEPES, and 5.5 glucose (pH 7.4, adjusted with NaOH). The patch pipettes (borosilicate glass; 1–3 MΩ) were filled with a standard pipette solution containing (in mM) 110 potassium aspartate, 20 KCl, 8 NaCl, 1 MgC1_2_, 1 CaC1_2_, 10 EGTA, 4 Na_2_ATP, and 10 HEPES (pH 7.2, adjusted with KOH). APs were elicited at 1 Hz by 2 ms to 5 ms current pulses applied through the patch pipette. RMP, APA, V_max_, and APD_20_, APD_50_, and APD_90_ were obtained from the last 30 seconds of 180 APs and averaged.

*I*_K_ — including *I*_peak_, *I*_to_, and *I*_ss_ — were assessed using a protocol of 500 ms voltage steps in 10 mV increments between –60 mV and +50 mV from a holding potential of –80 mV. Na^+^ current was inactivated with a 100 ms conditioning voltage step to –40 mV from the holding potential. *I*_CaL_ was blocked with 100 μM CdCl_2_ in Tyrode solution.

For transmembrane currents recording, atrial fibroblasts were bathed in a standard solution containing (in mM) 150 NaCl, 6 CsCl, 1 MgCl_2_, 5 CaCl_2_, 10 glucose, and 10 HEPES (pH 7.4, adjusted with NaOH). The pipettes solution contained (in mM) 20 CsCl, 100 cesium aspartate, 1 MgCl_2_, 10 HEPES, 4 Na_2_ATP, 10 EGTA, and 0.08 CaCl_2_ (pH 7.2, adjusted with CsOH). Cells were held at a potential of 0 mV, and ramps from –100 mV to 100 mV with a duration of 400 ms were applied at a frequency of 0.1 Hz.

### Intracellular Ca^2+^ measurement.

Intracellular Ca^2+^ was measured as described previously (10). Briefly, both atrial fibroblasts and myocytes were loaded with 2 μM Fluo-4/AM (Molecular Probes) for 30 minutes at 37°C. With the Enspire Multimode Plate Reader (PerkinElmer), cells in 96-well plates were illuminated at 488 nm, and ﬂuorescence emissions at 525 nm were captured at 3-second intervals. Relative changes in Ca^2+^ influx stimulated by 300 nM GSK1016790A are presented as (F/F0) or fold changes (ΔF/F0), respectively. F represents fluorescence intensity, F0 represents the average fluorescence intensity before GSK1016790A stimulation, and ΔF represents the mean fluorescence intensity at the steady-state after drug stimulation minus F0. Cells were pretreated with 300 nM GSK2193874 for 60 minutes.

### Measurements of cell proliferation.

Atrial fibroblasts proliferation was measured using a FITC- bromodeoxyuridine (BrdU) cell proliferation Detection Kit (KeyGEN BioTECH) following the manufacturer’s instructions. Cells (5000–10,000 cells/well) were seeded in 96-well culture plates and deprived overnight in FBS-free DMEM. BrdU was added at a final concentration of 30 μM.

### Statistics.

Values were expressed as the mean ± SEM. Data were analyzed using a 2-tailed unpaired Student’s *t* test or a 1-way ANOVA for multiple groups followed by a Bonferroni’s post hoc test using OriginPro 2018 (OriginLab Corporation). The incidence of atrial ectopy or fibrillation was compared with a χ^2^ test. Only results with *P* < 0.05 were considered statistically significant.

### Study approval.

All animal experiments were performed in accordance with the *Guide for the Care and Use of Laboratory Animals* (National Academies Press, 2011) and were approved by the Animal Research Ethics Committee of Tongji Medical College, Huazhong University of Science and Technology, Wuhan, China. For human samples, all patients gave written consent to the study. Human study was performed in accordance with the ethical guidelines of the 1975 Declaration of Helsinki and was approved by the ethics review committee of Union Hospital, Tongji Medical College.

## Author contributions

YD, LC, and QL designed the experiments. JL performed ex vivo optical mapping and patch clamp experiments. CQ, ZZ, KL, and SZ prepared the SP rats and performed in vivo electrophysiology experiments. JL and QW isolated atrial myocytes and atrial fibroblasts. NZ and QD included the patients. JL, QW, CQ, and NZ analyzed the data. JL and YD wrote the manuscript. LC and QL provided critical review of the data and advice throughout the research. YD supervised and provided funding for the project. All authors reviewed the manuscript.

## Supplementary Material

Supplemental data

## Version 1 10/29/2020

In-Press Preview

## Version 2 12/03/2020

Electronic publication
