# Supplementary material for: TRPV4 blockade suppresses atrial fibrillation in sterile pericarditis rats
Source: JCI Insight. 2020 Dec 3;5(23):e137528. doi: 10.1172/jci.insight.137528 (PMC7714415; doi:10.1172/jci.insight.137528)
Supplement: Supplemental data [file jciinsight-5-137528-s158.pdf]

Supplemental Table 1 Clinical features of patients studies

	SR	AF
Patient number, n	16	12
Gender, male/female	13/3	12/0
Body mass index, Kg/m^2	27.9 ± 0.8	28.5 ± 0.7
Smoking, n	7	7
Diabetes, n	5	6
Hyperlipidemia, n	8	6
Cardiac infarct, n	2	3
PCI, n	2	2
Cerebral infarct, n	3	1
AVD/MVD, n	3/4	1/3
LVEF, %	52.5 ± 3.2	53.8 ± 3.6
LA, mm	44.2 ± 4.2	45.6 ± 4.4
β-blocker, n	9	8
RAS inhibitor, n	9	10
Diuretics, n	11	12

Results are expressed as the mean ± SEM. SR, sinus rhythm as control; AF, atrial fibrillation; PCI indicates percutaneous coronary intervention; AVD, aortic valve disease; MVD, mitral valve disease; LVEF = left ventricular ejection fraction; LA = left atrium; RAS, renin-angiotensin system

Supplemental Table 2. Evaluation of Surface ECG Parameters and Transesophageal Recording.

	pre-operation			post-operation		
	sham	vehicle	GSK2193874	sham	vehicle	GSK2193874
HR, times/min	462.00 ± 5.60	456.67 ± 13.53	466.50 ± 13.43	456.17 ± 12.06	458.67 ± 10.69	458 ± 12.85
PR, ms	46.99 ± 0.71	43.59 ± 1.27	44.77 ± 1.89	46.00 ± 1.78	43.89 ± 2.44	43.95 ± 1.12
threshold, mA	1.81 ± 0.15	1.78 ± 0.19	1.78 ± 0.27	1.75 ± 0.17	2.00 ± 0.224	1.97 ± 0.17
QRS, ms	14.60 ± 0.66	16.23 ± 1.05	14.60 ± 0.37	15.31 ± 1.16	18.46 ± 1.83	17.22 ± 1.05
QT, ms	54.60 ± 0.90	54.40 ± 1.23	52.34 ± 1.97	51.98 ± 2.36	55.08 ± 1.13	52.74 ± 0.77
WCL, ms	82.37 ± 2.59	72.48 ± 12.26	83.15 ± 2.07	82.44 ± 1.93	86.37 ± 1.83	83.51 ± 2.07
CSNRT, ms	36.40 ± 4.17	38.00 ± 6.00	32.67 ± 2.40	39.50 ± 6.65	47.50 ± 12.28	33.97 ± 2.47
AVERP120, ms	71.57 ± 2.18	74.33 ± 2.78	74.50 ± 4.67	74.50 ± 2.19	78.43 ± 1.63	74.83 ± 2.44
AVERP110, ms	71.86 ± 2.76	73.83 ± 3.39	74.75 ± 4.55	73.17 ± 2.08	78.43 ± 2.72	75.83 ± 2.50
AVERP100, ms	72.00 ± 2.75	73.17 ± 3.66	73.75 ± 4.23	73.33 ± 1.91	79.14 ± 2.70	76.50 ± 2.85

ECG and transesophageal recording from sham (n = 10), vehicle (n = 8), and GSK2193874 (n = 6). Results are expressed as the mean ± SEM. HR indicates heart rate; WCL, Wenckebach cycle length; CSNRT, corrected sinus node recovery time; AVERP, atrioventricular nodal refractory period.

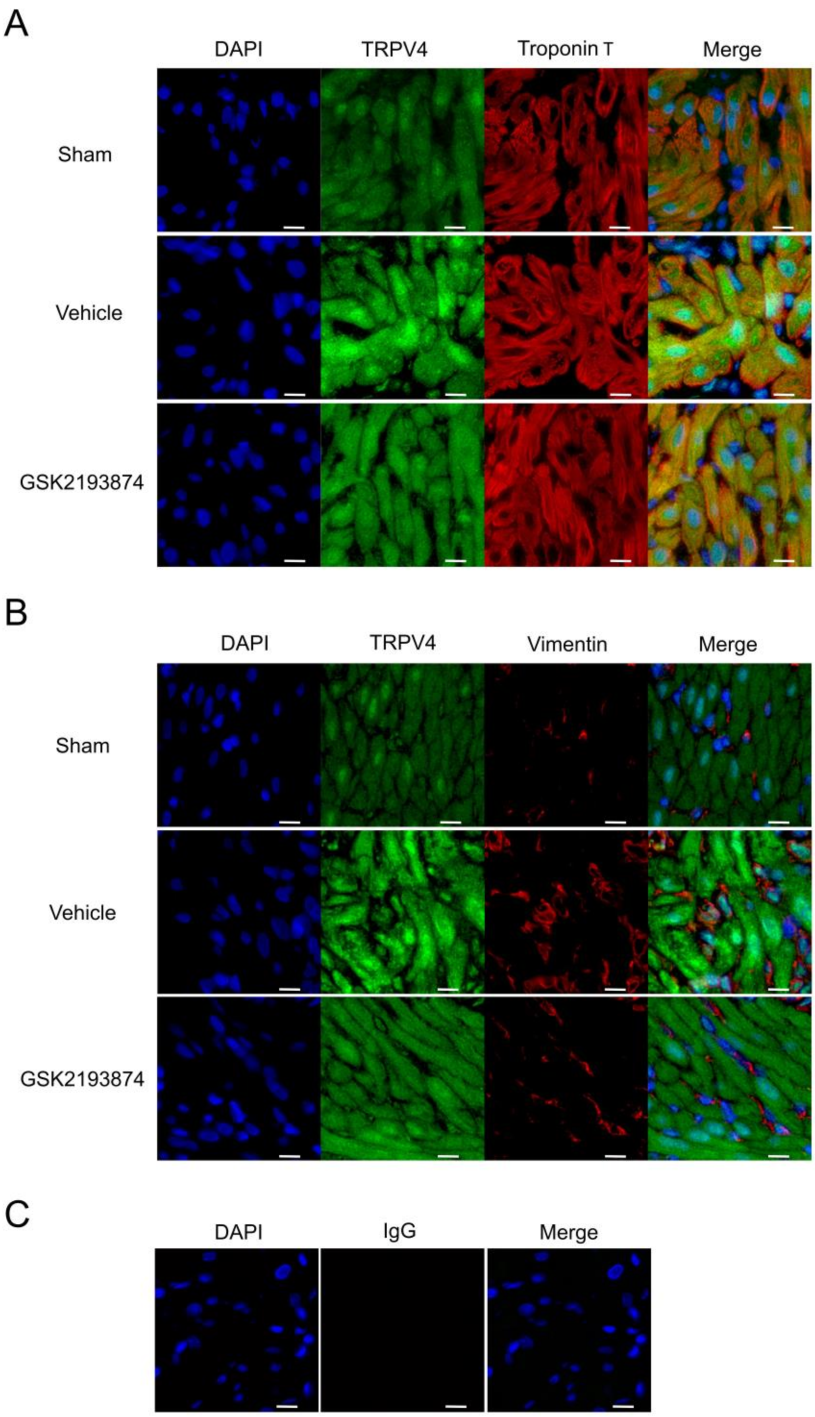

Supplemental Figure 1. Localization of TRPV4 in the rat atria by immunofluorescence. Representative images of double-immunofluorescent labeling of TRPV4 and Cardiac Troponin T (A)/vimentin (B) in the sham, vehicle and GSK2193874 groups. Negative control was performed using specific IgG instead of TRPV4 antibody (C). Scale bar: 10 μm.

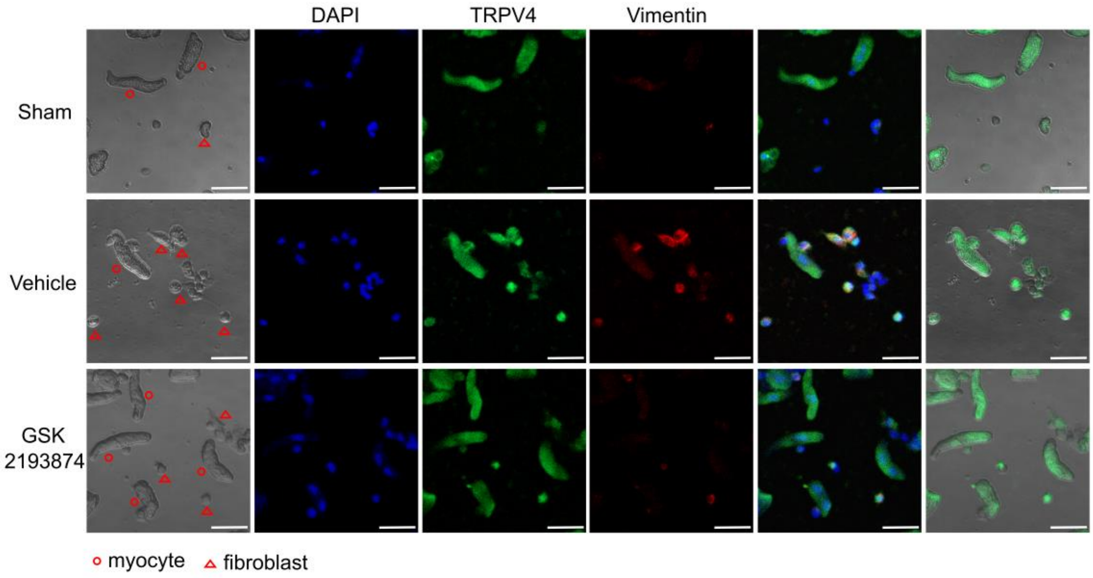

Supplemental Figure 2. Localization of TRPV4 in the isolated atrial myocytes and fibroblasts by immunofluorescence. Representative images of immunostaining of atria from the sham, vehicle and GSK2193874 groups. Red= Vimentin (a fibroblast marker), Green= TRPV4 and Blue= DAPI to stain for nuclei. Scale bar: 50 μm.

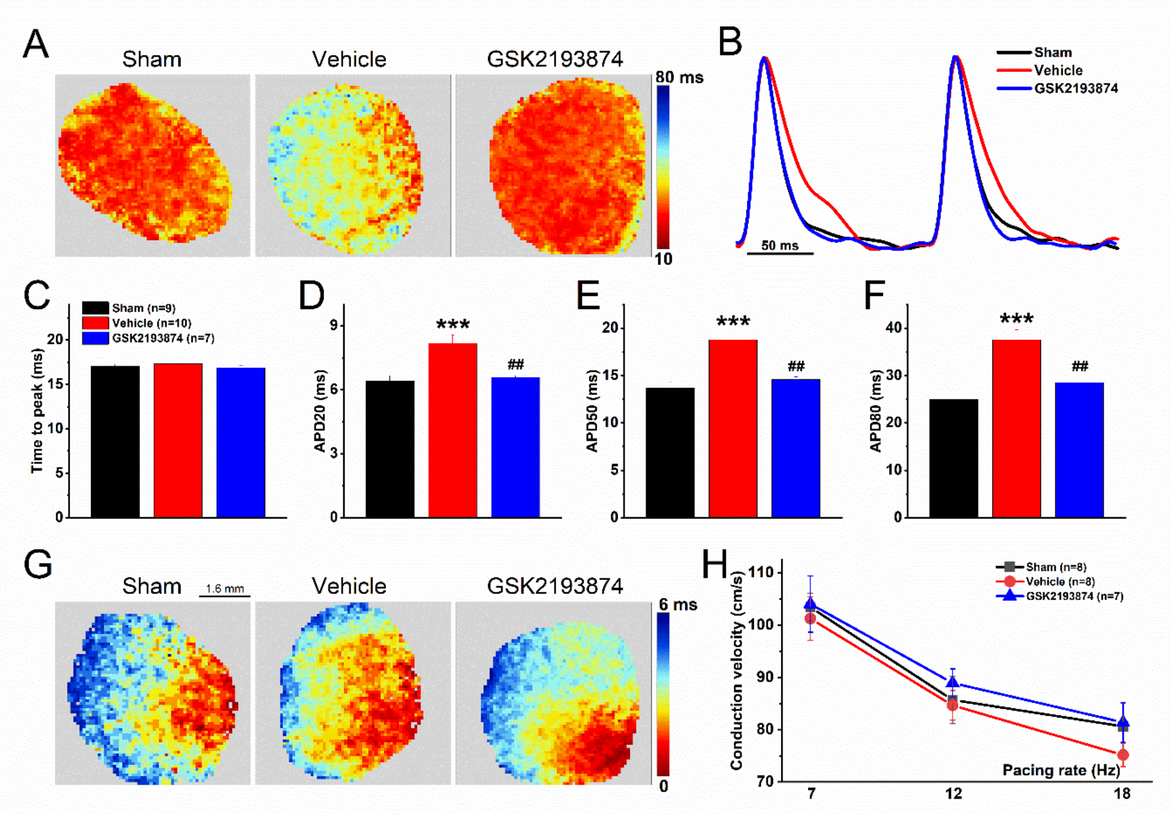

Supplemental Figure 3. Optically recorded atrial action potential (AP) and activation maps. Typical atrial AP duration at 80% repolarization (APD_{80}) maps (A) and corresponding AP traces (B) at a pacing rate of 7 Hz recorded from the sham, vehicle, and GSK2193874 groups. Quantification of time to peak (C), APD_{20} (D), APD_{50} (E), and APD_{80} (F); sham n = 9; vehicle n = 10; GSK2193874 n = 7. Representative atrial activation maps (G) and quantification of conduction velocity (H) in the three groups; sham n = 8; vehicle n = 8; GSK2193874 n = 7. Statistical analyses: A one-way ANOVA with Bonferroni's post-hoc test (C, D, E, and F);***P < 0.001 vs. sham; ##P < 0.01 vs. vehicle. Results are expressed as the mean ± SEM.

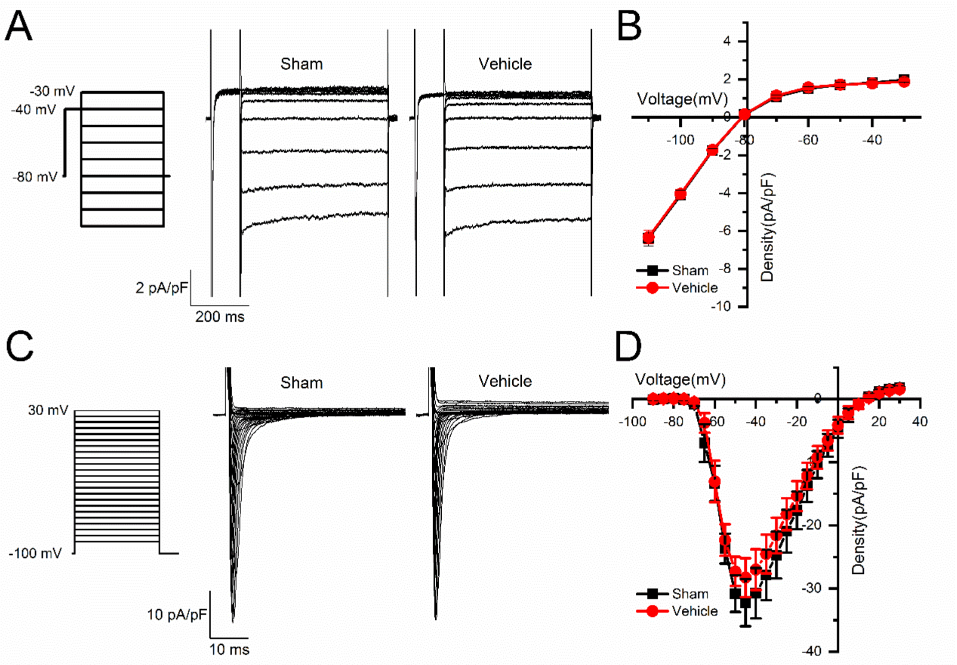

Supplemental Figure 4. Inward rectifier K^+ current (I_{K1}) and sodium current (I_{Na}) recorded from isolated atrial myocytes from sham and vehicle-treated rats. A. Original I_{K1} current traces. Voltage clamp protocol is shown in the left panel. B. Mean current-voltage (*I*–V) curves for I_{K1}. sham n=25 myocytes/ 5 rats; vehicle n =41 myocytes/ 6 rats. The measurement method of I_{K1} is the same as that of voltage-gated K^+ currents (I_K), except for the protocol of 500-ms voltage steps in 10-mV increments between -110 mV and -30 mV from a holding potential of -80 mV. C. Original I_{Na} current traces. Voltage clamp protocol is shown in the left panel. D. Mean *I*-V curves for I_{Na}. For I_{Na} recordings, the bath solution containing (in mM): 20 NaCl, 50 tetraethylammonium-Cl, 67 CsCl, 10 HEPES, 10 glucose, 1 $MgCl_2$, 1 $CaCl_2$, 0.1 $CdCl_2$ (pH adjusted to 7.4 with CsOH). The pipette solution containing (mM): 5 NaCl, 5ATP-Mg, 125 CsCl, 10 EGTA, 10 HEPES (pH adjusted to 7.4 with CsOH). Sham n = 13 myocytes / 5 rats; vehicle n = 11 myocytes / 4 rats. Statistical analyses: A one-way ANOVA with Bonferroni's *post-hoc test* (B and D); Results are expressed as the mean ± SEM.

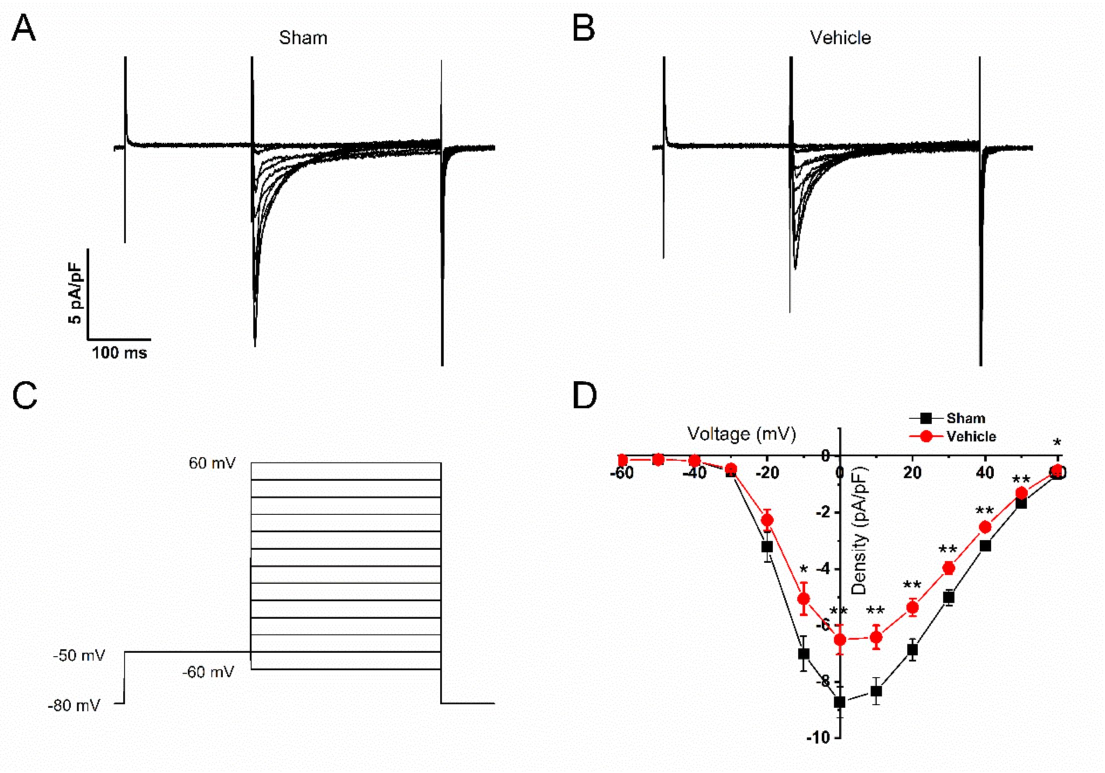

Supplemental Figure 5. L-type calcium current ($I_{Ca,L}$) recorded from isolated atrial myocytes from sham and vehicle-treated rats. Original $I_{Ca,L}$ current traces from sham (A) and vehicle group (B), voltage clamp protocol (C) and mean *I*–V curves (D). For $I_{Ca,L}$ recordings, the bath solution containing (in mM): 140 tetraethylammonium-Cl, 10 HEPES, 5.5 glucose, 1 $MgCl_2$, 2 $CaCl_2$ (pH adjusted to 7.4 with CsOH). The pipette solution containing (mM): 130 CsCl, 10 EGTA, 4 Na_2ATP, 6.6 sodium phosphocreatine, 10 HEPES, 0.01 GTPs, and 1 $MgCl_2$ (pH adjusted to 7.2 with CsOH). sham n = 31 myocytes / 4 rats; vehicle n = 28 myocytes / 4 rats. Statistical analyses: A one-way ANOVA with Bonferroni's *post-hoc test* (D). Results are expressed as the mean ± SEM.

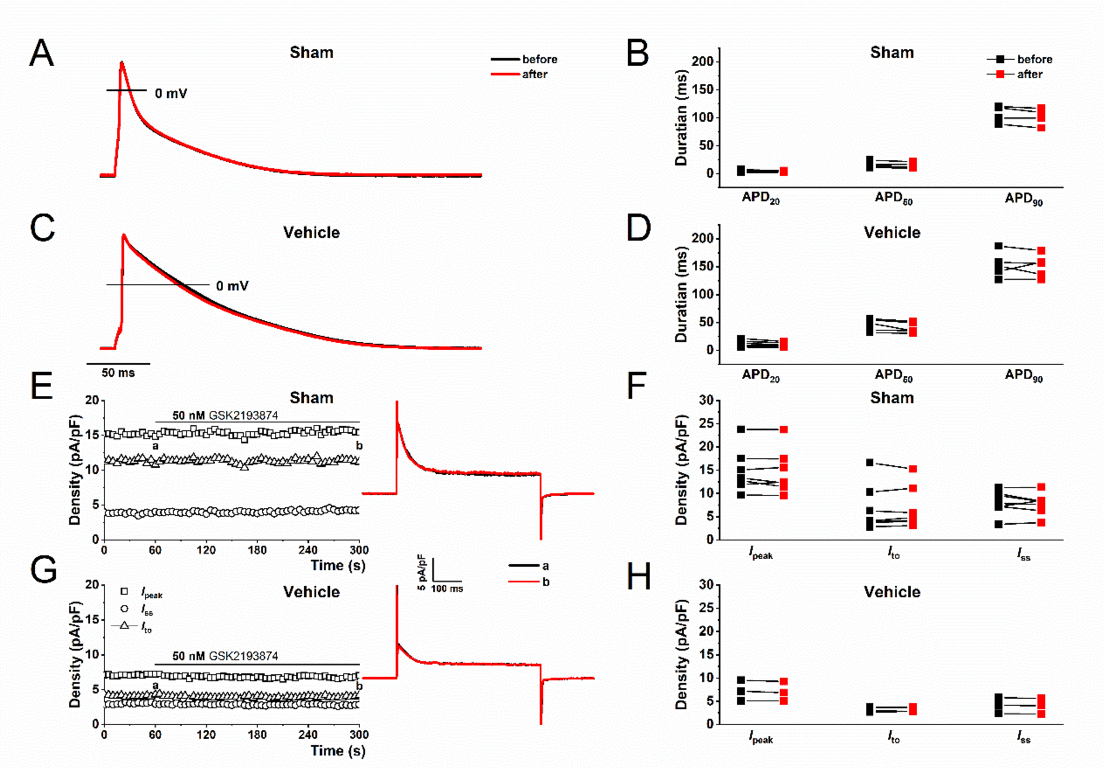

Supplemental Figure 6. Acute effect of TRPV4 blocking on atrial electrical remodeling. Representative action potentials (APs) recorded from isolated atrial myocytes of sham group (A) and vehicle group (C), before and after the application of GSK2193874 (50 nM). Quantification of APD_{20}, APD_{50}, and APD_{90} from isolated atrial myocytes of sham group (B) and vehicle group (D), before and after the application of GSK2193874; sham n = 4 myocytes / 2 rats; vehicle n = 5 myocytes / 3 rats. Time course of outward voltage-gated K^+ currents (I_K) at 50 mV (left panel) and corresponding current trace taken at time points a, b (right panel) with the application of GSK2193874, in isolated atrial myocytes of sham group (E) and vehicle group (G). Quantification of I_{peak}, I_{ss}, and I_{to} from isolated atrial myocytes of sham group (F) and vehicle group (H), before and after the application of GSK2193874; sham n = 7 myocytes / 3 rats; vehicle n=3 myocytes / 2 rats. Results are expressed as the mean ± SEM.

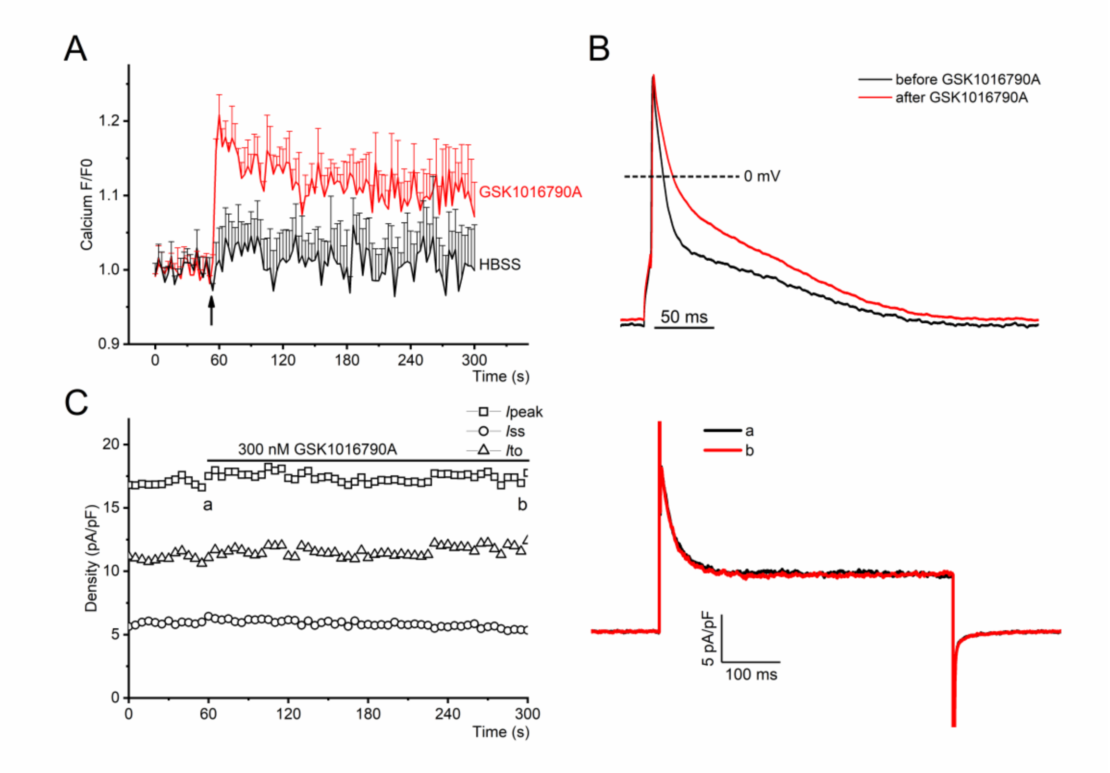

Supplemental Figure 7. Acute effect of TRPV4 agonist GSK1016790A on the Ca^{2+} influx and electrophysiological properties of atrial myocytes. A. Representative time course of the changes in $[Ca^{2+}]_i$ induced by 300 nM GSK1016790A or HBSS in atrial myocytes from rats. B. Representative APs recorded from isolated atrial myocytes, before and after the application of 300 nM GSK1016790A. C. Time course of outward voltage-gated K^+ currents at 50 mV (left panel) and corresponding current trace taken at time points a, b (right panel) with the application of 300 nM GSK1016790A in isolated atrial myocytes.

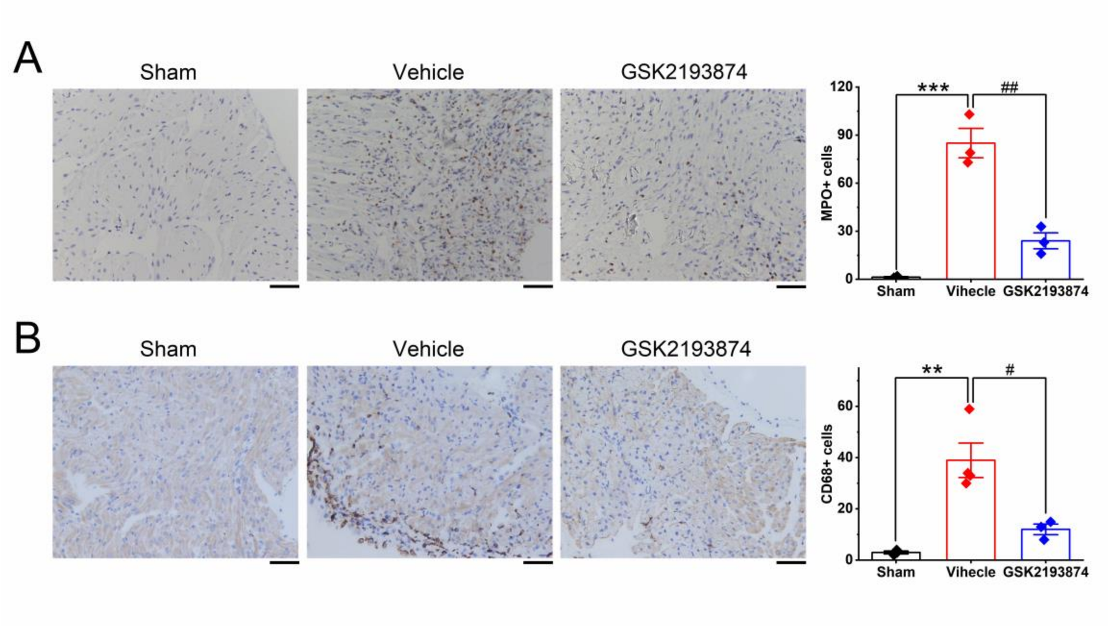

Supplemental Figure 8. The blockage of TRPV4 attenuates atrial immune cell infiltration in SP rats. A. Representative images of atrial MPO staining and quantification. Five visual fields are taken in each sample, and the number of immune cells from these fields is quantified with ImagePro 6.0 software and is averaged to make a statistical analysis. sham n = 3; vehicle n = 3; GSK2193874 n = 3. Scale bar: 50 µm. B. Representative images of atrial CD68 staining and quantification. sham n = 3; vehicle n = 4; GSK2193874 n = 3. Scale bar: 50 µm. Statistical analyses: A one-way ANOVA with Bonferroni's post-hoc test; **P < 0.01, ***P < 0.001 vs. sham; #P < 0.05, ##P < 0.01 vs. vehicle. Results are expressed as the mean ± SEM.

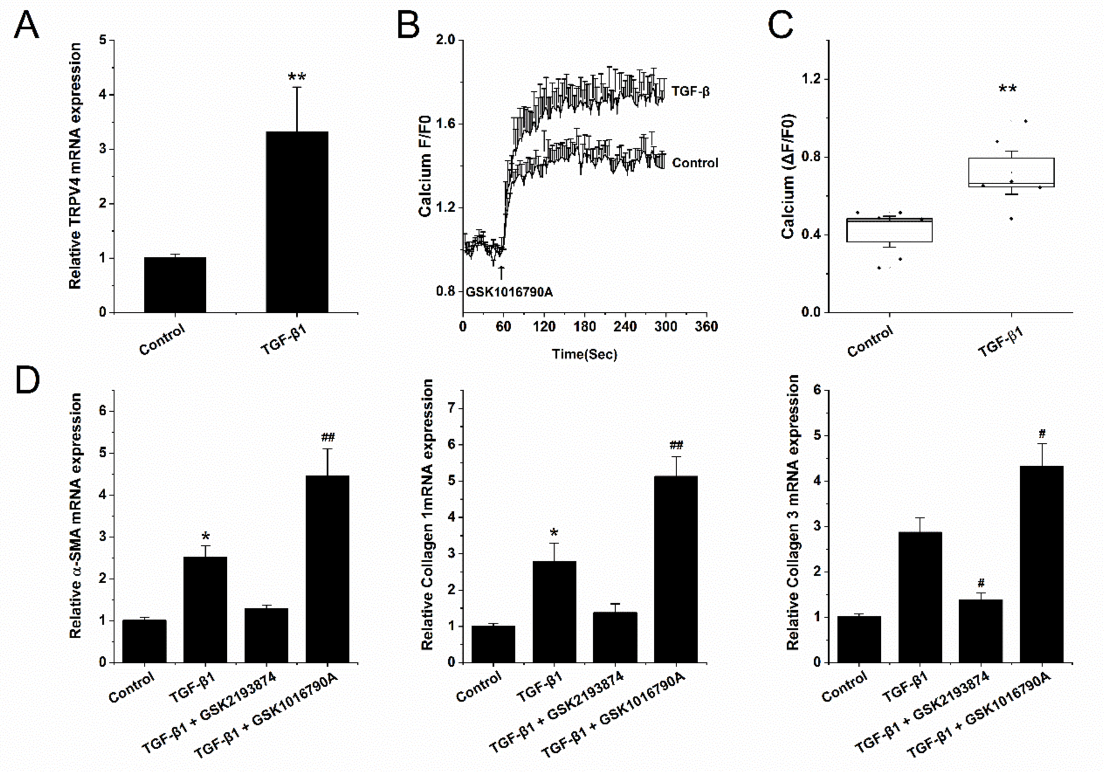

Supplemental Figure 9. TRPV4 is involved in TGF-β1-induced atrial fibroblasts differentiation. Cells were isolated from sham rats and stimulated with TGF-β1 in the presence or absence of TRPV4 antagonist/agonist GSK2193874/GSK1016790A. A. The mRNA expression of TRPV4. Representative time course (B) of the changes in $[Ca^{2+}]_i$ and quantification (C) induced by GSK1016790A. D. The mRNA expression of α-SMA, collagen-1, and collagen-3 by real-time PCR. n = 6 / group, each in triplicate; Statistical analyses: A *t*-test (A and C) and a one-way ANOVA with Bonferroni's *post-hoc test* (D);*P < 0.05, **P < 0.01 vs. control; #P < 0.05, ##P < 0.01 vs. TGF-β1. Results are expressed as the mean ± SEM.
